# Can improved canopy light transmission ameliorate loss of photosynthetic efficiency in the shade? An investigation of natural variation in *Sorghum bicolor*

**DOI:** 10.1093/jxb/erab176

**Published:** 2021-04-29

**Authors:** Nikhil S Jaikumar, Samantha S Stutz, Samuel B Fernandes, Andrew D B Leakey, Carl J Bernacchi, Patrick J Brown, Stephen P Long

**Affiliations:** 1 Institute for Genomic Biology, University of Illinois at Urbana-Champaign, Urbana, IL 61801, USA; 2 Department of Plant Biology, University of Illinois at Urbana-Champaign, Urbana, IL 61801, USA; 3 Department of Crop Sciences, University of Illinois at Urbana-Champaign, Urbana, IL 61801, USA; 4 USDA ARS Global Change and Photosynthesis Research Unit, Urbana, IL 61801, USA; 5 Department of Plant Sciences, University of California at Davis, Davis, CA 95616, USA; 6 Lancaster Environment Centre, University of Lancaster, Lancaster LA1 4YQ, UK; 7 University of Essex, UK

**Keywords:** C_4_ photosynthesis, crop canopy architecture, food security, leaf form, quantum efficiency, stomata, water use efficiency

## Abstract

Previous studies have found that maximum quantum yield of CO_2_ assimilation (Φ _CO2,max,app_) declines in lower canopies of maize and miscanthus, a maladaptive response to self-shading. These observations were limited to single genotypes, leaving it unclear whether the maladaptive shade response is a general property of this C_4_ grass tribe, the Andropogoneae. We explored the generality of this maladaptation by testing the hypothesis that erect leaf forms (erectophiles), which allow more light into the lower canopy, suffer less of a decline in photosynthetic efficiency than drooping leaf (planophile) forms. On average, Φ _CO2,max,app_ declined 27% in lower canopy leaves across 35 accessions, but the decline was over twice as great in planophiles than in erectophiles. The loss of photosynthetic efficiency involved a decoupling between electron transport and assimilation. This was not associated with increased bundle sheath leakage, based on ^13^C measurements. In both planophiles and erectophiles, shaded leaves had greater leaf absorptivity and lower activities of key C_4_ enzymes than sun leaves. The erectophile form is considered more productive because it allows a more effective distribution of light through the canopy to support photosynthesis. We show that in sorghum, it provides a second benefit, maintenance of higher Φ _CO2,max,app_ to support efficient use of that light resource.

## Teaser

In dense crop stands, the lower leaves of sorghum show a maladaptive loss of photosynthetic efficiency, lowering potential yield. Screening multiple genotypes shows selection for more upright leaves to protect against this loss.

## Introduction

A major component of crop yield increases over the last century has been increased planting density (Duvick, 2005; Lee and Tolenaar, 2007). The ability to tolerate high planting density depends on a number of morphological traits, including leaf inclination angle (Williams *et al.*, 1968; Tian *et al.*, 2011; ). In maize, for example, erectophile plants, with small inclination angles relative to the vertical (i.e. highly erect leaves), are associated with higher grain yield than planophiles (i.e. with drooping leaves and high leaf inclination angles: Pendleton *et al.*, 1968; Lambert and Johnson, 1978; Lee and Tolenaar, 2007; Lauer *et al.*, 2012). Associations between erect leaf angle and higher yield are also found in rice (Sinclair and Sheehy, 1999; Kumagai *et al.*, 2014), wheat (Richards *et al.*, 2019), sugarcane (DaSilva and DeCosta, 2012), and soybean (Jin *et al.*, 2003). In sorghum [*Sorghum bicolor* (L.) Moench], the world’s fifth most important cereal crop (Rai *et al.*, 1999), considerable variation exists for leaf inclination angle (Xin *et al.*, 2009) and for further increases in planting density (Xin *et al.*, 2015). A number of genes influencing this trait have been identified (Truong *et al.*, 2015; Xin *et al.*, 2015), and leaf angle is being incorporated as a direct target for improvement through breeding or synthetic biology. However, the causes of the association between erect leaf angle and yield improvement may not yet be fully understood, particularly in canopies of C_4_ crops of the Andropogoneae. This tribe includes the highly productive crops: sorghum, sugarcane (*Saccharum officinarum* L.), maize, and the key bioenergy crop giant miscanthus (*Miscanthus×giganteus* Greef et Deuter). Therefore there is considerable scope for further work both to improve leaf erectness through selection and to understand better the mechanistic basis for why leaf erectness is linked to improved carbon accumulation by sorghum and related crops.

The most obvious and best studied mechanism by which leaf erectness influences productivity is through directly optimizing light distribution through the canopy (Tian *et al.*, 2011; Gitz *et al.*, 2015). The relationship between light intensity and carbon assimilation is hyperbolic. The uppermost leaves of crop canopies typically experience high light, and gradually approach their asymptotic maximal photosynthetic value (*A*_sat_); in contrast, in heavily shaded lower leaves, photosynthesis is light limited and responds linearly to an increase in light. Photosynthetic capacity under strictly light-limited conditions is described by the initial slope of the photosynthetic light–response curve; that is, the maximum quantum yield of carbon assimilation (Φ _CO2,max,abs_ on an absorbed light basis, Φ _CO2,max,app_ on an incident light basis). Therefore, increasing light penetration through the canopy is expected to allow more light interception by the lower canopy levels, allowing for a net increase in carbon assimilation at the canopy scale (Srinivasan and Long, 2017). Other mechanisms have also been hypothesized: erect leaves may allow for more efficient shedding of heat (Hunt *et al.*, 2018), while the greater leaf area index (LAI) allowed by erectophile canopies may serve as a nitrogen storage sink (Sinclair and Sheehy, 1999). In addition, the degree to which increased light penetration through erectophile canopies might affect leaf photosynthetic traits, and the extent to which these effects might help explain the observed association between leaf erectness and yield, remains an ongoing topic of research, especially in C_4_ species. Previous models of erectophile versus planophile sorghum canopies attributed yield improvements entirely to the direct effects of improved light distribution, and did not take into account potential changes in leaf photosynthetic traits that might accompany changes in canopy architecture (Gitz *et al.*, 2015; Truong *et al.*, 2015). However, the light environment is known to strongly influence the leaf photosynthetic machinery, particularly in the context of self-shading where leaves that develop under high light are gradually subjected to progressively heavier shading as stem and leaf tissue grow above them. Measuring the effects of self-shading on photosynthetic traits might therefore allow for improved predictive power of canopy photosynthesis models, and for a better mechanistic understanding of how increased leaf erectness in sorghum might result in improved productivity.

The extent to which lower canopy leaves may be able to acclimate to progressively heavier shading over their life span constitutes a significant knowledge gap, particularly in the context of the Andropogoneae. In systems such as deciduous forests, lower canopy leaves spend their entire life span in low light, and are known to acclimate to these conditions. Based on ecological theory and experimental evidence (e.g. Syvertsen, 1984), shaded leaves are expected, and found, to invest in thinner leaves, more chlorophyll, a decreased Chl *a*/*b* ratio, and reduced levels of Calvin cycle enzymes. These changes result in lower respiration (*R*_D_), lower light-saturated photosynthetic rate (*A*_sat_), unchanged maximal quantum yield of assimilation on an absorbed light basis (Φ _CO2,max,abs_: Mooney and Gulmon, 1979; Long *et al.*, 1993; Hikosaka and Terashima, 1995), and higher quantum yield on an incident-light basis (Φ _CO2,max,app_). However, such ‘classical’ models of shade adaptation are based largely on experiments where leaves experience constant low- or high-light environments. They may not be applicable to situations such as fast-developing crop canopies, in which leaves that begin their lives in high light are subjected to progressively increased self-shading as younger leaves shade older ones, and where shading may affect the microenvironment, in particular light quality such as blue to red ratios. A few studies have found evidence that some C_3_ species, under conditions of progressive self-shading, can partially acclimate to shading and potentially maintain high Φ _CO2,max,abs_. In *Beaucarnia stricta* and *Davallia bullata*, lower canopy leaves exhibit similar Φ _CO2,max,abs_ to upper canopy leaves (Long *et al.*, 1993). A similar ability to maintain high Φ _CO2,max,abs_ in the lower canopy is seen in wheat (*Triticum aestivum* L.) crops and wild oat (*Avena fatua* L.; Beyschlag *et al.*, 1990). Upper canopy leaves in *Dryobalanops aromatica* C.F. Gaertn also experience increased self-shading as they age, and acclimate by trading carbon fixation capacity for increased light-harvesting capacity, while maintaining the same Φ _CO2,max,abs_ as they age (Ishida *et al.*, 1999). However, evidence from two species of the Andropogoneae indicates that they may not be able to acclimate effectively to self-shading. Lower canopy leaves of maize and giant *Miscanthus* were found to have 14–15% and 27–29% lower Φ _CO2,max,abs_, respectively, compared with upper canopy leaves (Pignon *et al.*, 2017). These results were shown to be effects of shade rather than leaf age, based on comparisons between exposed leaves at the southern edge of stands, with similarly aged shaded leaves within the interior of the stands (Collison *et al.*, 2020). Quantum yield decline in the lower canopy, in maize and miscanthus, might be linked to the difficulties of coordinating CO_2_ uptake by phosphoenolpyruvate carboxylase (PEPC) in the mesophyll and by Rubisco in the bundle sheath. Bundle sheath leakiness (ϕ) is known to increase with declining light (Kromdijk *et al.*, 2008; Tazoe *et al.*, 2008), which could lead to decreases in the productivity and efficiency of photosynthesis in the lower canopy. However, previous studies on the photosynthetic effects of self-shading in the Andropogoneae have considered only one or two genotypes of the species examined, and studies in sugarcane have found a similar effect in one genotype, but no effect on quantum yield in another (Marchiori *et al.*, 2014). Therefore, it remains a topic for further inquiry, whether quantum yield decline under self-shading is broadly present within the germplasm of a species and across the Andropogoneae.

In a set of field studies across 4 years, the effect of self-shading on photosynthetic performance in lower canopy sorghum leaves was studied. Carbon assimilation, electron transport, stomatal conductance, and activity of three key C_4_-specific photosynthetic enzymes were compared between upper and lower canopy leaves, within a genetically diverse range of accessions varying widely in canopy architecture and thereby in the degree of self-shading. Accessions with erect leaves and high light transmission through the canopy are henceforth referred to as ‘erectophile’ and those with low leaf erectness as ‘planophile’. In the final year of the study, bundle sheath leakiness in erectophile and planophile accessions was also compared. The objectives of this study were 3-fold: (i) to test the hypothesis that quantum yields would decrease in the lower canopy of a wide range of sorghum genotypes; (ii) to test the hypothesis that erectophile genotypes, that allow improved light penetration through the canopy, would show a smaller loss of photosynthetic efficiency in the lower canopy leaves; and (iii) to test whether bundle sheath leakiness would increase and concentrations of C_4_ enzymes needed to support the light-saturated rate of photosynthesis would decline in the lower canopy leaves. All three hypotheses were supported. The erectophile form is known to allow a more effective distribution of light, thereby supporting higher canopy-level photosynthesis. Here we show that a secondary benefit is that it allows the lower leaves to maintain higher quantum yields, so further boosting canopy carbon gain and productivity.

## Materials and methods

### Planting conditions

A biomass sorghum diversity panel previously reported in genetic studies by Valluru *et al.* (2019) and dos Santos *et al.* (2020) was used for this study. In summary, the panel was composed of 869 photoperiod-sensitive sorghum accessions that do not flower at our study location and are referred to as biomass or fiber accessions. The panel consisted of 3% bicolor, 18% caudatum, 22% durra, 21% guinea, 1% kafir, and 35% mixed-ancestry accessions. Geographic collection sites ranged worldwide, although the most common sources were Mali and Ethiopia. The diversity panel was planted in 2016 and 2017 at two sites: Savoy, IL (Fisher Farm in 2016: 40.02°N, 88.24°W and Maxwell Farm in 2017: 40.04°N, 88.24°W) and Urbana, IL (Energy Farm in all years: 40.06°N, 88.19°W). Soils at the Savoy sites are fine-silty mixed mesic Typic Endoaquolls and at the Urbana site fine montmorillonitic, mesic Aquic Argiudolls. Each accession was planted in a four-row plot (one plot per site) randomized in an augmented block design, as described in dos Santos *et al.* (2020). Plots consisted of a row length of 3.0 m, with 1.5 m alleys and 0.76 m between rows within a plot. Target density was ~270 368 plants ha^−1^. Seeds were treated with fluxofenim safener and mefenoxam fungicide before planting. Plots were planted in late May using a four-row planter attached to a tractor. (*S*) metolachlor herbicide was applied during the growing season. Plants were harvested in early October using a four-row small plot harvester (Kemper Head 5830: John Deere, Moline, IL, USA). Over the period 15 May–30 September, total precipitation was 569 mm in 2016, 309 mm in 2017, 572 mm in 2018, and 436 mm in 2020: mean temperature was 23.2 °C in 2016, 22.2 °C in 2017, 23.1 °C in 2018, and 21.9 °C in 2020; and daytime highs exceeded 32 °C on 25 d in 2016, 26 d in 2017, 32 d in 2018, and 24 d in 2020. The core photosynthetic traits were measured at both sites in 2016 and 2017. Canopy extinction coefficients were measured in 2018 and 2020, leaf absorptance in 2018, and bundle sheath leakiness in 2020. Levels of C_4_ mesophyll cycle enzymes were measured in 2017 and Rubisco content was measured in 2020.

### Screening for leaf inclination angle

All accessions were screened for leaf inclination angle, manually with protractors, between 5 and 22 July 2016 and between 26 June and 14 July 2017. Leaf inclination angle is defined here as the angle of the leaf at the point of insertion relative to the vertical. The assumption, subsequently tested, was that leaf inclination angle would be a proxy for canopy light transmission. Leaf inclination angle was assessed at the base of the lamina, from the ligule and point of attachment to the sheath. Both the youngest fully expanded leaf (i.e. the youngest showing ligule emergence) and the oldest fully green leaf were measured for three plants of each of the 869 accessions at each site. Accessions with extreme phenotypes—the 40 with the lowest and 40 with the highest leaf inclination angles relative to the vertical—were identified. Because inclination angles of the youngest fully expanded leaf and oldest fully green leaf were moderately correlated in 2016 (*r*^2^=0.32, *P*<0.0001) and in 2017 (*r*^2^=0.60, *P*<0.0001), only the youngest fully expanded leaf was used to identify accessions of interest. Erectophile accessions are defined as having leaf inclinations relatively close to the vertical (low inclination angle at the youngest fully expanded leaf) and planophile accessions as having relative inclinations close to the horizontal (high inclination angle). From this subset of 80 extreme phenotypes, 10 erectophiles and eight planophiles were selected at random for photosynthetic trait analysis in 2016, after removing those with high coefficients of variation (>0.33), and 12 erectophiles and nine planophiles were selected following the same procedure in 2017. Across the two years, 18 erectophile and 17 planophiles were represented, since four erectophile accessions overlapped between the two years. The accessions in 2018 were taken from the erectophile and planophile ends of the 2017 screening, and mostly overlapped with the accessions that had been chosen for photosynthesis in 2017; as three lines were missing due to inadequate seed quantity (two planophile and one erectophile), three alternative lines were substituted, comprising 18 erectophiles and 17 planophiles. In 2020, the measurement of canopy light profiles involved 24 accessions (12 erectophile and 12 planophile) comprising most of the 2016 and 2017 accessions. Since not all of the previous years’ lines were available due to inadequate seed quantity, five others (all erectophiles) were chosen randomly from the 2017 angle screening and added to the study to compensate for missing accessions

### Light environment

To verify that leaf erectness influenced light distribution through the canopy, photosynthetic photon flux density (PPFD, herein referred to as ‘*Q*’) in the canopy was measured with a 1 m line quantum sensor (Decagon Devices: Pullman, WA, USA) to provide a spatially averaged photon flux at two heights within the canopy: at the youngest fully expanded leaf near the top of the canopy and at the lowest fully green leaf. Measurements of *Q* were made on 7 August 2016 and 12 August 2017. In addition, full canopy light profiles were measured on 15 July and 12 August 2018, on the same set of accessions that had been chosen for photosynthetic sampling in 2017. Here, light was measured at the top of the canopy, defined as height=1, and then at heights of 0.75, 0.5, 0.25, and 0.1 of the distance from the ground (0) to the top of the canopy. Extinction coefficients (*k*) were determined as follows:


$$\begin{array}{l} Q_{Z}=Q_{0}e^{-kZ} \end{array}$$

where *Q*_0_ = incident light above the canopy and *Z* = depth into the canopy.

On 14 July 2020, extinction coefficients were measured again, this time based on six data points, at the canopy surface and then at relative distances of 0.8, 0.6, 0.4, 0.2, and 0.1 from the ground (0) to the top of the canopy (1).

### Photosynthetic light–response curves for accessions from the diversity panel

Leaf photosynthetic traits were measured with portable infrared CO_2_/water vapor gas exchange systems each with a modulated chlorophyll fluorometer integrated into its leaf cuvette (LI-6400 XT, LI-COR, Lincoln, NE, USA). The Savoy site was sampled from 4 to 10 August 2016 and from 21 to 25 August 2017: the Urbana site was sampled from 12 to 18 August 2016 and from 10 to 18 August 2017. For each accession, sun leaves and shade leaves were collected at dawn. The cut ends were placed in water and immediately re-cut under water to allow the water column to re-fill, following Pignon *et al.* (2017). This procedure avoided confounding photosynthetic capacity with diurnal changes in leaf water potential and temperature, as well as any transient photoinhibition or feedback inhibition from accumulation of non-structural carbohydrates over the course of the day. The youngest fully expanded leaf was used to typify ‘sun’ leaves and the oldest fully green leaf (i.e. with no sign of senescence or significant damage) to typify ‘shade’ leaves.

Cut leaves were transported to the laboratory where leaves were clamped into the cuvette of the gas exchange system. Cuvette conditions were 30 °C block temperature, *Q*=2000 µmol m^–2^ s^−1^, and [CO_2_]=400 µmol mol^−1^, with a water vapor pressure deficit of 1.27–1.91 kPa. Once steady state (variation of <3% over 1 min) was achieved, the response of photosynthesis to light was determined. Gas exchange and fluorescence parameters were measured at *Q*=2400, 2000, 1600, 1200, 800, 600, 400, 300, 250, 200, 150, 100, 80, 60, 40, and 20 µmol m^−2^ s^−1^. The multiphase flash fluorescence (MPF) protocol (Loriaux *et al.*, 2013) was used to estimate maximum chlorophyll fluorescence in the light (*F*_m_′) from which PSII efficiency (Φ _PSII_) and the ratio of variable to maximal fluorescence in the light (*F*_v_′ /*F*_m_′) was calculated. The 900 ms long MPF intensity was 8000 μmol m^−2^ s^−1^ with a 20% phase 2 ramp. To calculate photosynthetic traits on an absorbed light basis, leaf absorptance (α) was determined using an integrating sphere and scanning spectrometer (JAZ, Ocean Optics: Largo, FL, USA) coupled with the SpectraSuite software, on parallel leaf samples collected from the same accessions in 2018. This allowed the conversion of measured incident light (*Q*) to light absorbed by the leaf (*Q*_abs_).

Light–response curves were fit by non-linear regression (NL procedure in STATA 15.1: StataCorp, College Station, TX. USA). Light–response curves were modeled as a non-rectangular hyperbola, with Equations 2, 3, and 4 describing assimilation, electron transport, and stomatal conductance, respectively.


$$\begin{array}{l} A={\left\{A_{sat}+{\;\Phi \;}_{CO2,max,app}\;Q-[{\left(A_{sat}+\Phi_{CO2,max,app}\right)}^{2}-4A_{sat}\;\Phi_{CO2,max,app\;\;}\theta Q]\right\}}^{0.5}/[2\theta_{A}]-R_{D} \end{array}$$

$$\begin{array}{l} J={\left\{J_{max}+{\;\Phi }_{J}\;Q\;-\;[{\left(J_{max}+\Phi_{J}\right)}^{2}-4J_{max}\;\Phi_{J\;}\theta Q]\right\}}^{0.5}/[2\theta_{J}] \end{array}$$

$$\begin{array}{l} G_{S}={\left\{G_{S,max}+{\;\Phi }_{G}\;Q[{\left(G_{S,max}+\Phi_{G}\right)}^{2}-4G_{S,max}\;\Phi_{G\;}\theta G]\right\}}^{0.5}/[2\theta_{G}] \end{array}$$

Here *A*_sat,_*J*_sat_, and *G*_S,sat_ represent light-saturated values, and Φ _CO2, max,abs_, Φ _J_, and Φ _G_ represent light-limited quantum yields, namely initial slopes of assimilation, electron transport, and stomatal conductance against light, respectively. The parameter *R*_D_ represents light-adapted respiration, *G*_0_ represents stomatal conductance at zero light, and θ is a convexity parameter. To gain better precision for our estimates of quantum yield of gas exchange (Φ _CO2,max,abs_) and quantum yield of electron transport (Φ _J_), linear regressions were separately conducted on the initial portion of the curve; that is, *Q*<100–150 µmol m^−2^ s^−1^. The exact range, for each plant, was chosen to optimize *r*^2^ for this initial linear portion (Long *et al.*, 1993). Initial slopes for the *A* versus *Q* response were calculated on an incident light basis (apparent quantum yield, Φ _CO2,max,app_). Dividing this value by leaf absorptance (α) gave quantum yield on an absorbed light basis (Φ _CO2,max,abs_). Quantum yield of electron transport (Φ _J_) was estimated from ΦPSII measurements, using Equation 5 and assuming a partition of 0.4 between PSII and PSI (Edwards and Baker, 1993),


$$\begin{array}{l} J=0.4\;\alpha Q\left(\Phi PSII\right) \end{array}$$

Constraints for the model were *R*_D_ >0.3, *G*_0_ >0.01, and 1>θ>0.01.

*Q* values from field measurements described above were combined with parameters extracted from light–response curves, to estimate leaf-level carbon assimilation under ambient light conditions (*A*_ambient_) as well as analogous estimates for electron transport (*J*_ambient_) and intrinsic water use efficiency (IWUE_ambient_). Here, ‘ambient’ refers to the light environment that sun and shade leaves were actually experiencing in the field at midday, on sunny days in late summer after full canopy closure. The import of these estimates is to simulate carbon assimilation, electron transport, and water use efficiency under field conditions, and to gain information about how the effects of changes in leaf-level photosynthetic traits and canopy light environments might compound one another. To calculate, *A*_ambient_, *J*_ambient_, and IWUE_ambient_ for shade leaves of a particular accession (e.g. ‘Doronko’), the light intensity values measured in the field for upper and lower canopy *Q* were substituted in Equations 2, 3, and 4, respectively, using the parameters estimated from light–response curves (Φ _CO2_, *A*_sat_, etc.) for ‘Doronko’ shade leaves, as well as measured values of absorptance (α).

### Leakiness (ϕ) in upper and lower canopy leaves

Upper and lower leaves (youngest fully expanded leaf and oldest fully green leaf) from erectophile and planophile accessions were excised from the plants in the field at dawn: the cut ends were immediately placed in water and cut again under water to allow the water column to re-fill. Leaves were cut between 05.30 h and 06.30 h from 18 to 26 August 2020. Cut leaves were taken to the lab and remained with cut ends continuously under water, in the dark, until photosynthesis was measured.

Leaves were placed in a controlled-environment plant growth cabinet (Gen 1000, Conviron Controlled Environments, Winnipeg, Canada) with a *Q* of ~700 μmol m^−2^ s^−1^ and 35 °C for ~5 min before being placed in the large flat leaf chamber of a LI-6800 (LICOR Biosciences) located in the growth cabinet. Leaf temperature was set to 35 °C, *Q* to 2200 μmol m^−2^ s^−1^, [CO_2_] to 400 μmol mol^−1^, and chamber relative humidity to 50% with 21% [O_2_]. Leaves were allowed to acclimate while the rate of photosynthesis and stomatal conductance increased to a steady state, requiring 20–35 min. After ~30 min, irradiance was decreased to 80 µmol quanta m^-2^ s^-1^. Once photosynthesis stabilized under 80 μmol quanta m^−2^ s^−1^, the LI-6800 was matched and the plant was allowed to photosynthesize for another 30 min. The LI-6800 was coupled to a tunable diode laser absorption spectroscope (TDL; model TGA 200A, Campbell Scientific, Inc., Logan, UT, USA) measuring ^12^CO_2_, ^13^CO_2_, and δ ^13^C (Bowling *et al.*, 2003; Pengelly *et al.*, 2010; Ubierna *et al.*, 2013, 2017 ). Input gases (N_2_ and O_2_) were mixed using mass flow controllers (Omega Engineering Inc., Norwalk, CT, USA) linked to a datalogger and a voltage output module (CR1000 and SDM-CV04, Campbell Scientific, Inc.). A portion of the N_2_/O_2_ stream was passed to the LI-6800 while the remainder was used to correct for drift in the TDL over the course of the measurements. The TDL was calibrated by diluting a 10% CO_2_ tank in the N_2_/O_2_ stream to produce four different [CO_2_] of the same isotopic composition (Tazoe *et al.*, 2009; Pengelly *et al.*, 2010; Ubierna *et al.*, 2013). The measurement sequence consisted of a zero [CO_2_] and three [CO_2_], a calibration tank with a known [^12^CO_2_], [^13^CO_2_], and δ ^13^C composition, followed by the LI-6800 reference, and a sample leaf chamber. The TDL measured each of the six gas streams for 20 s. The LI-6800 was set to autolog every 140 s, once per TDL measurement cycle. The TDL was connected to the LI-6800 reference using the reference port on the back of the LI-6800 head. The port on the front of the LI-6800 was used for measuring the [^12^CO_2_], [^13^CO_2_], and δ ^13^C composition of the leaf chamber air.

### Calculations of photosynthetic discrimination (∆ ^13^C) and estimation of bundle sheathleakiness (ϕ) under high light

Online photosynthetic discrimination (∆ ^13^C_obs_) was calculated according to Evans *et al.* (1986):


$$\begin{array}{l} {\;\Delta \;}^{13}C_{obs}=\frac{1000\xi (\delta^{13}C_{samp}-\delta^{13}C_{ref})}{1000+\delta^{13}C_{samp}-\xi (\delta^{13}C_{samp}-\delta^{13}C_{ref})} \end{array}$$

where δ ^13^C_samp_ and δ ^13^C_ref_ are the carbon isotope compositions of the leaf chamber and reference air in the LICOR and where ζ is:


$$\begin{array}{l} \xi =\frac{C_{ref}}{\left(C_{ref}-C_{samp}\right)} \end{array}$$

where C_ref_ and C_samp_ are the CO_2_ concentrations of dry air entering and exiting the leaf chamber, respectively, measured by the TDL.

The enzyme-limited estimate of leakiness, including the ternary effect (*t*) (Farquhar and Cernusak, 2012), under high irradiance was estimated using the model proposed by Ubierna *et al.* (2013):


$$\begin{array}{l} \phi_{HL}=\frac{\frac{\left(1-t\right){\;\Delta \;}^{13}C_{obs}C_{a}-a^{{\;}^{\prime }\;}(C_{a}-C_{i})}{(1+t)C_{i}}-b_{4}^{{\;}^{\prime }\;}+\frac{e^{\prime }R_{m}}{A+0.5R_{D}}}{b_{3}^{{}^{\prime }}-s+e^{{\;}^{\prime }\;}\left(\frac{R_{m}}{A+0.5R_{D}}-\frac{R_{D}}{A+R_{D}}\right)} \end{array}$$

where, *C*_a_ and *C*_i_ are the ambient and intercellular CO_2_ partial pressure, respectively. The fractionation during leakage from the bundle sheath cells (*s*) is 1.8‰, b′ _3_ (29‰) is the Rubisco fraction, and b′ _4_ (–4.7‰) is the net fractionation by CO_2_ dissolution, hydration, and PEPC activity at 35 °C (Farquhar, 1983; Henderson *et al.*, 1992; von Caemmerer *et al.*, 2014). *R*_D_ is leaf mitochondrial respiration in the light, *R*_m_ (*R*_m_=0.5*R*_D_) is the rate of mesophyll cell respiration in the light, and *A* is the rate of photosynthesis. The fractionation during decarboxylation including measurement artifacts (*e′*) is estimated according to Wingate *et al.* (2007):


$$\begin{array}{l} e^{{\;}^{\prime }}=e+\;\delta^{13}C_{ref}-\delta^{13}C_{gatm} \end{array}$$

where *e* is the respiratory fractionation during decarboxylation, δ ^13^C_ref_ is the isotopic signature of the CO_2_ entering the LICOR reference, and δ ^13^C_gatm_ is the isotopic signature of the CO_2_ where the plants are grown, –8‰ in our study.

The ternary effect (*t*) (Farquhar and Cernusak, 2012) takes into account the carbon isotope discrimination on the rate of CO_2_ assimilation and is calculated as:


$$\begin{array}{l} t=\frac{\left(1+a^{\prime }\right)E}{2g_{ac}^{t}} \end{array}$$

where, *E* is the rate of transpiration and *g*^*t*^_ac_ is the total conductance to CO_2_ diffusion including boundary layer and stomatal conductance (von Caemmerer and Farquhar, 1981) and *a*′ denotes the combined fractionation factor through the leaf boundary layer and stomata:


$$\begin{array}{l} a^{\prime }=\frac{a_{b}\left(C_{a}-C_{L}\right)+a_{s}(C_{L}-C_{i})}{\left(C_{a}-C_{i}\right)} \end{array}$$

where *C*_L_ is the leaf surface CO_2_ partial pressure, *a*_b_ (2.9‰) is the fractionation occurring through diffusion in the boundary layer, and *a*_s_ (4.4‰) is the fractionation due to diffusion in air (Evans *et al.*, 1986).

### Estimation of bundle sheath leakiness (ϕ) in low light.

For our estimates of ϕ under low light, we assumed that mesophyll conductance was infinite (*C*_i_=*C*_m_), based on the modeling of Ubierna *et al.* (2011) which demonstrated that the effects of *g*_m_ on C_4_ photosynthetic discrimination calculations under low light were small. We also assumed a constant bundle sheath conductance (*g*_bs_) following Brown and Byrd (1993). Leakiness under light-limited conditions was then calculated according to Ubierna *et al.* (2013):


$$\begin{array}{l} \phi_{LL}=\frac{1}{C_{i}}\frac{\;\Delta \;{}_{}^{13}C_{obs}\left(1-t\right)C_{a}-a^{\prime }\left(C_{a}-C_{i}\right)-(1-t)C_{i}b_{4}}{\left(1+t)(b_{3}-s\right)} \end{array}$$

where, *b*_3_ is the ^13^C fractionation during carboxylation by Rubisco including respiration and photorespiration, and *b*_4_ is the net fractionation by CO_2_ dissolution, hydration, and PEPC including respiratory fractionation:


$$\begin{array}{l} b_{3}=b_{3}^{{\;}^{\prime }\;}-\frac{e^{\prime }R_{D}}{V_{c}}-\frac{fV_{o}}{V_{c}} \end{array}$$

and


$$\begin{array}{l} b_{4}=b_{4}^{{\;}^{\prime }\;}-\frac{e^{{\;}^{\prime }\;}R_{m}}{V_{p}} \end{array}$$

where, *b′*_3_ (30‰) and *f* (11.6‰) are the ^13^C fractionation during carboxylation by Rubisco (Roeske and O’Leary, 1984) and ^13^C fractionation during photorespiration (Lanigan *et al.*, 2008), respectively. The changes in net fractionation by CO_2_ dissolution, hydration, and PEP carboxylation including respiratory fraction (Farquhar, 1983; Henderson *et al.*, 1992), *b′*_4_, with temperature were calculated at 35 °C according to von Caemmerer *et al.* (2014):


$$\begin{array}{l} b_{4}^{\prime }=\frac{-9.483\times 1000}{273+\left(T{}^{\circ }C\;\right)}+23.89+2.2 \end{array}$$

*V*_c_, *V*_o_, and *V*_p_ are the rate of Rubisco carboxylation, Rubisco oxygenation, and PEP carboxylation, respectively (von Caemmerer, 2000; Ubierna *et al.*, 2013):


$$\begin{array}{l} V_{c}=\frac{A+R_{D}}{1-\frac{\gamma^{*}O_{bs}}{C_{bs}}} \end{array}$$

$$\begin{array}{l} V_{o}=\frac{V_{c}-A-R_{D}}{0.5} \end{array}$$

$$\begin{array}{l} V_{p}=\frac{xJ_{t}}{2} \end{array}$$

where γ* is half the reciprocal of Rubisco specificity at 35 °C (0.000291) and *x* is the fraction of *J*_t_ allocated to the C_4_ cycle, 0.4–40% of the total electron flux is used for the C_4_ portion of the cycle (von Caemmerer, 2000; Ubierna *et al.*, 2011). *O*_bs_ and *C*_bs_ are the the partial pressure of oxygen and CO_2_ in the bundle sheath cells, respectively:


$$\begin{array}{l} O_{bs}=\frac{\alpha A}{0.047g_{bs}}+O_{m} \end{array}$$

$$\begin{array}{l} C_{bs}=\frac{(\gamma^{*}O_{bs})(\frac{7}{\text{3}}\left(A+R_{D}\right)+\frac{(1-x)J_{t}}{3}}{\frac{(1-x)J_{t}}{3}-(A+R_{D})} \end{array}$$

where, *g*_bs_ is the bundle sheath conductance in sorghum (0.00113 mol m^−2^ s^−1^; Brown and Byrd, 1993) and *O*_m_ is the oxygen partial pressure in the mesophyll cell, assumed to be equal to the partial pressure of oxygen in the intercellular airspace (mesophyll conductance is infinite). We modeled *J*_t_, the total electron transport rate as a function of photosynthetic rate, using the equations for the light-limited C_4_ photosynthesis model of von Caemmerer (2000):


$$\begin{array}{l} J_{t}=\frac{-II+\sqrt{II^{2}-4\times III\times I}}{2\times III} \end{array}$$

where,


$$\begin{array}{l} I=\left(1+\frac{R_{D}}{A}\right)\times \left(R_{m}-g_{bs}C_{m}-\frac{7g_{bs}\times \gamma^{*}O_{m}}{3}\right)+\left(R_{D}+A\right)\times \left(1-\frac{7\alpha \gamma^{*}}{3\times 0.047}\right) \end{array}$$

$$\begin{array}{l} II=\frac{1-x}{3}\left[\frac{g_{bs}}{A}\left(C_{m}-\frac{R_{m}}{g_{bs}}-\gamma^{*}O_{m}\right)-1-\frac{\alpha \times \gamma^{*}}{0.047}\right]-\frac{x}{2}\left(1+\frac{R_{D}}{A}\right) \end{array}$$

and


$$\begin{array}{l} III=\frac{x-x^{2}}{6A} \end{array}$$

where α is the fraction of PSII activity in the bundle sheath (0 in sorghum).

### Enzyme activity assays

Activities of three key C_4_-specific photosynthetic enzymes, pyruvate orthophosphate dikinase (PPDK), PEPC, and malate dehydrogenase (MDH), were measured in crude extracts from sun and shade leaves collected from the Urbana site on 4 September 2017. Samples of ~60 mg each were collected, flash-frozen, and hand-ground under liquid nitrogen. Proteins were then extracted, following the protocol of Pittermann and Sage (2000), in a buffer of 50 mM HEPES (pH 8.2), 1% polyvinylpyrrolidone, 0.14% (w/v) BSA, 5 mM DTT, 10 mM MgCl_2_, 10% (v/v) glycerol, 1 mM EDTA, and one protease inhibitor cocktail tablet per 50 ml of buffer, for 20 min at room temperature on a shaker. Extracts were centrifuged at full speed for 60 s and separated into aliquots for the analyses.

PPDK was assayed using the method of Pittermann and Sage (2000) with the modifications of Wang *et al.* (2008). Assay buffer was comprised of 50 mM Tris (pH = 7.7), 7 mM DTT, 4 mM glucose-6-phosphate, 10.5 U ml^–1^ porcine MDH, 1 U ml^−1^ microbial PEPC, 5 mM MgCl_2_, 1.5 mM EDTA, 10 mM NaHCO_3_, 5 mM NH_4_Cl, 2.5 mM K_2_HPO_4_, 0.3 mM NADH, 2 mM ATP, and 1 U ml^−1^ myokinase. An aliquot of 20 µl of leaf extract was added to 270 μl of buffer and the reaction was initiated with addition of 30 µl of 20 mM sodium pyruvate.

MDH was assayed following a modification of Kumar *et al.* (2000). The reaction buffer comprised 100 mM Tris–HCl (pH = 7.8), 30 mM DTT, 0.5 mM EDTA, 0.3 mM NADPH, 8 mM MgCl_2_, 10 mM NaHCO_3_, 1 U ml^−1^ PEPC, and 2 mM glucose-6-phosphate. The reaction was initiated with addition of 30 µl of 20 mM PEP to a mixture of 20 µl of leaf extract and 270 µl of buffer, following incubation for 20 min to achieve activation of the enzyme.

PEPC was assayed following Sales *et al.* (2018). The assay buffer included 50 mM Tris–HCl (pH = 7.8), 5 mM MgCl_2_, 10 mM NaHCO_3_, 0.3 mM NADH, 10.5 U ml^−1^ porcine MDH, 1 mM EDTA, and 2 mM glucose-6-phosphate. An aliquot of 20 µl of leaf extract was added to 270 μl of buffer and the reaction was initiated with addition of 30 µl of 20 mM pyruvate. In all three assays, the reaction rate was measured as change in absorbance of NADH or NADPH at 340 nm using an extinction coefficient of 6.22 M^−1^ cm^−1^. Measurements were done at 30 °C. PEPC was assayed over the course of 10 min and extrapolated to *t*=0, using the observed exponential decrease in reaction rate with time, to account for end-product inhibition: the other two enzymes were assayed over the course of 10 min. All chemicals were from Sigma-Aldrich (St. Louis, MO, USA).

Since Rubisco was not measured in 2017 due to limited sample material, tissue samples from upper and lower canopy leaves of erectophile and planophile accessions were collected on 12 August 2020. Protein extractions were performed as described above. Rubisco content was assayed via a competitive ELISA, using the Plant Rubisco ELISA Kit (Aviva Systems Biology, San Diego, CA, USA). Protein extracts (50 µl) were incubated along with 50 µl of anti-Rubisco antibody solution, in well plates pre-coated with Rubisco, for 60 min at 37 °C. Wells were then washed and incubated with a secondary antibody conjugated to horseradish peroxidase (100 µl), for 60 min at 37 °C. Finally, wells were thoroughly washed again and incubated with 90 µl of tetramethylbenzidine solution for 20 min at 37 °C. Absorbance was read at 450 nm and compared with a standard curve.

### Statistical analysis

Photosynthetic data were analyzed via two distinct methods. The first was an analysis of covariance, to explore whether canopy type (erectophile versus planophile) modulated the effect of canopy position, after controlling for height. The effects of year×site were combined to give an ‘environment’ term, so that there were a total of *n*=4 ‘environments’. Individual accessions were only replicated at two sites per year, and estimating effects of accessions would be difficult. Therefore, we omitted the effect of accession from our model, treating accessions as individual observations within the broader population of erectophile or planophile types, and analyzed data using plant height as a covariate:


$$\begin{array}{l} y=\mu +type+height+environment+type\\ \times environment+position+type\times position \end{array}$$

Here, *y* represents each photosynthetic parameter, μ is the grand mean, ‘type’ represents erectophile or planophile canopy structure (two levels), ‘height’ represents plant height used as a covariate, ‘environment’ is the combined effect of year×type (four levels), ‘position’ represents high or low canopy position (two levels), and ‘type×position’ interaction (four levels) would indicate that that the degree of self-shading, as hypothesized, modulates the effect of canopy position.

Enzyme activity and leakiness data were collected at only a single site, in one year, thus effects of environment and accession could not be estimated. The model here, therefore, omitted the environment term and accession terms, and analyzed data as a split plot design with ‘type’ (erectophile versus planophile) and ‘position’ as the split plot factor. Height was not included in 2020 as height estimates had not been concluded at the time of submission. Failures in the harvester meant that not all accessions were harvested in each year at each site; therefore, data from both sites were combined. The model was the following, with ‘year’ representing the effect of calendar year.


$$\begin{array}{l} y=\mu +type+height+year \end{array}$$

Additionally, data were analyzed using a paired design, comparing upper and lower canopy leaves within each accession to estimate the effect of canopy position and the interaction of type×position. In the paired design, each accession was treated as a single individual, pooling across the two sites and across the two years, in cases where the accession was sampled in both years. The difference between values for upper and lower canopy leaves (Δ*y*, e.g. Δ*A*_sat_=*A*_sat,upper_–*A*_sat,lower_) within each plot was used as the response variable. The Δ*y* values for erectophile and planophile accessions were compared to estimate the effect of ‘position × type’, and the combined Δ*y* values for both classes (erectophiles + planophiles) were compared against 0 to estimate the effect of ‘position’. For ‘position × type’, a significant effect shows, as above, that the degree of self-shading modulates the effect of canopy position. In addition, since leaf angle could be considered as a continuous rather than a categorical variable, each variable was subjected to linear regression against leaf angle, treating each accession as a data point. Linear regression was done separately for 2016 and 2017.

Homogeneity of variance was assessed by Hartley’s test. To improve homogeneity of variance for ANOVA and planned contrasts, values for electron transport under ambient light conditions (*J*_ambient_), assimilation rate under ambient conditions (*A*_ambient_), the ratio of electron transport quantum yield to assimilation quantum yield (Φ _J_/Φ _CO2_), intrinsic water use efficiency at ambient light (IWUE_ambient_), and stomatal conductance at saturating light (*g*_S,sat_) were cube-root transformed. For the same reason, quantum yield of electron transport (Φ _J_), PEPC activity, PPDK activity, and incident light (*Q*) were square-root transformed. All statistical analyses used the ANOVA procedure in STATA 15.1 (Stata Statistical Software: Release 15 StataCorp LLC, College Station, TX, USA).

## Results

### Screening for leaf angle and effects on light transmission in diverse accessions

**Fig. 1.**
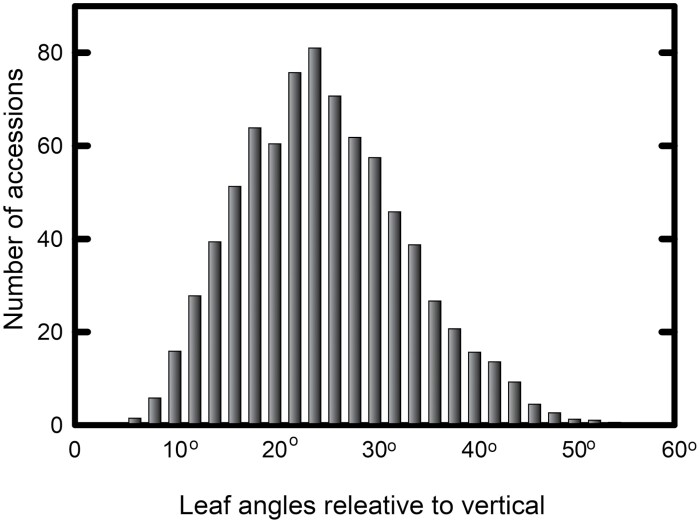
Measurement of the leaf inclination angle. Leaf inclination angle (relative to the vertical) for the youngest fully expanded leaf of 869 sorghum accessions, measured in July 2016 and July 2017 at two sites (Savoy, IL and Urbana, IL).

**Fig. 2.**
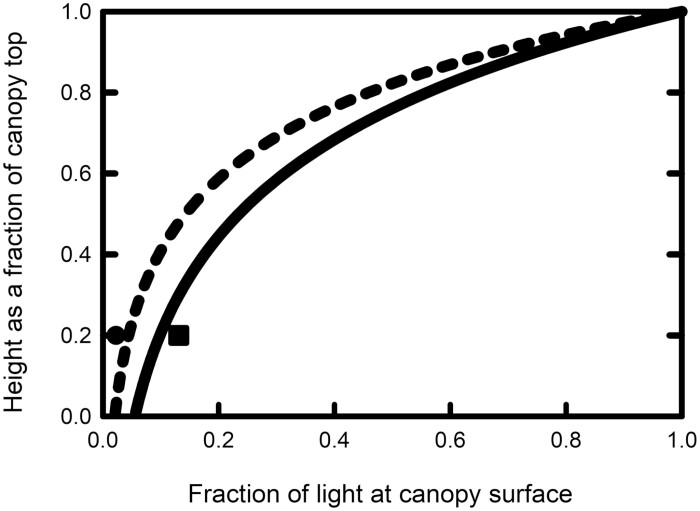
Fraction of incident light retained. Fraction of incident light (relative to full sunlight) retained, as a function of relative height from the top of the canopy to the ground (1.0=top of canopy, 0=ground surface) in sorghum accessions with small leaf inclination angles from the vertical (erectophile: solid line) and large leaf inclination angles (planophile: broken line). Light profiles were measured with a line quantum sensor in July and August 2018, averaged across the accession selected for photosynthesis measurements. In addition, light at one-fifth of the canopy height, approximating to the lowest fully green leaves, was measured in each year. The fraction of light remaining at this height averaged across the lines used for photosynthesis, for both study sites in July and August of each of 2016, 2017, and 2018, is given for the planophile (filled circle) and erectophile (filled square) accessions.

The biodiversity panel showed large variation in leaf inclination angle. Leaf angles for the youngest fully expanded leaf averaged 9–12° in ‘erectophiles’ and 44–48° for ‘planophiles’ in 2016, and 10–12° and 44–49°, respectively, in 2017. Distributions of leaf angles are shown in Fig. 1, with combined data from 2016 and 2017. Leaf erectness in all 4 years of the study had a profound impact on light penetration through the canopy. Averaged across the 4 years of measurements, the lowermost fully green leaf of erectophiles received 175% more light than in planophiles (Table 1). Extinction coefficients were 2.26 in erectophiles versus 3.15 in planophiles in July 2018 (*t*=2.32, *P*=0.029, df=24), 3.37 versus 4.42 in August 2018 (*t*=4.10, *P*<0.001, df=16: Fig. 2), and 2.60 versus 3.60 in July 2020 (*t*=2.32, *P*=0.029, df=24). The generally higher extinction coefficients in August compared with July 2018 reflect the taller and denser canopy structure achieved by this growth stage. Leaf angles for the accessions in 2016 and 2017 are given in Supplementary Tables S1 and S2, respectively. Parameters extracted from light profiles in 2018 are given in Supplementary Table S3, while light profiles measured in 2020 are given in Supplementary Table S4.

**Table 1.** Means (±SE), *r*^2^ for correlation against leaf inclination angle, Student’s *t*-values for planned contrasts, and *F*-values from ANOVA, for maximal light-saturated carbon assimilation (*A*_sat_), light-adapted respiration (*R*_D_), convexity of the light–response curve (θ), incident photosynthetically active radiation (*Q*), light-saturated stomatal conductance (*G*_S,sat_), water use efficiency at ambient light level in the field (IWUE_ambient_), and slope of the stomatal conductance versus light–response curve (Φ _G_), for sun leaves and shaded leaves of erectophile (E) and planophile (P) sorghum accessions

Variable:	*A*_sat_	*R*_D_	Θ_A_	*Q*	*g*_S,sat_	IWUE_ambient_	Φ_g_
Treatment	µmol m^−2^ s^−1^		–	µmol m^−2^ s^−1^	mol m^–2^ s^−1^	μmol mol^−1^	mol mmol^–1^
P, Sun (2016)	30.1±2.6	1.5±0.1	0.56±0.05	1949±49	0.149±0.014	162±11	02.54±2.6
P, Shade (2016)	13.3± 1.5	0.9±0.1	0.54±0.03	108±12	0.061±0.010	62±7	1.68±1.6
Reduction (%)	56	40	4	94	58	61	34
E, Sun (2016)	27.7±2.0	1.6±0.1	0.66±0.05	1661±86*	0.139±0.005	166±7	3.46± 6.9
E, Shade (2016)	15.6±1.4	1.1±0.2	0.52±0.03	300±29***	0.065*±*0.009	160±33***	4.36±1.39
Reduction (%)	43	33	23	82	52	4	–26
P, Sun (2017)	37.8±3.3	2.4±0.3	0.46±0.05	1690±29	0.168±0.018	189±8	2.00±1.6
P, Shade (2017)	19.6±1.8	1.2±0.2	0.40±0.07	165±32	0.080±0.011	158±18	1.70±3.0
Reduction (%)	48	51	12	90	52	16	16
E, Sun (2017)	35.1±2.0	2.1±0.1	0.50±0.05	1674±24	0.146±0.009	185±5	2.15±1.1
E, Shade (2017)	23.9±1.5	1.7±0.1*	0.36±0.04	324±39**	0.104±0.009	179±12	1.87±1.7
Reduction (%)	32	18	28	81	28	3	13
**Correlation**							
**2016**	0.010	0.072	0.122	0.675**	0.006	0.184	0.198
**2017**	0.170	0.188*	0.002	0.237*	0.150	0.056	0.027
**Contrast**							
Position	–9.74***	–6.73***	–2.85*	–27.45***	–8.40***	–3.70***	0.84
Type× Position	–2.20*	–2.45*	1.65	–5.26***	–1.44	–3.21**	–1.44
**Source of variation**							
	*F*_12,143_	*F*_12,143_	*F*_12,143_	*F*_12,143_	*F*_12,143_	*F*_12,143_	*F*_12,143_
Environment	5.07**	4.91**	21.72***	0.93	0.51	0.61	2.13
Type	0.01	2.25	0.37	24.89***	0.27	13.28***	6.58*
Height	1.25	0.23	0.39	4.29*	0.09	1.37	0.55
Env×type	0.07	0.85	0.15	3.02*	1.17	1.50	0.65
Position	48.29***	21.69***	3.00	1905.20***	43.77***	19.71***	0.18
Type×Position	2.07	3.81*	1.03	42.14***	2.02	8.58***	1.36

IWUE_ambient_ was estimated at ambient light levels for each leaf at midday on a fully sunlit day in August; *A*_sat_ and *G*_S,max_ were measured under light-saturated conditions, while Φ _*G*_ was measured under light-limited conditions (PAR=0–150 μmol m^−2^ s^−1^). Eight planophile and 10 erectophile lines were measured in 2016; nine planophile and 12 erectophiles were measured in 2017. Correlation (*r*^2^) refers to the correlation of parameter values for lower canopy leaves against leaf angle, since changes in light distribution are expected to have the strongest effects in the lower canopy. Data are from a pair of field studies in 2016 and 2017 (Savoy, IL and Urbana, IL). In both years, each accession was replicated at two field sites. Symbols ‘*’, ‘**’, and ‘***’ denote statistical significance at a two-tailed probability; α=0.05, 0.01, and 0.001, respectively. This is for comparison of both erectophiles with planophiles at the same canopy position and within the same year, and for correlation, contrast, and sources of variation.

Leaf absorptance was, as expected, strongly affected by canopy position. Lower canopy leaves had significantly higher leaf absorptance than upper canopy leaves: this effect was larger in planophiles (4% increase, from 0.831–0.865 to 0.869–0.909) than in erectophiles (2% increase, from 0.832–0.847 to 0.845–0.870). Plant height did not vary between canopy types, averaging 365 cm in 2016 and 331 cm in 2017.

### Carbon assimilation

**Fig. 3.**
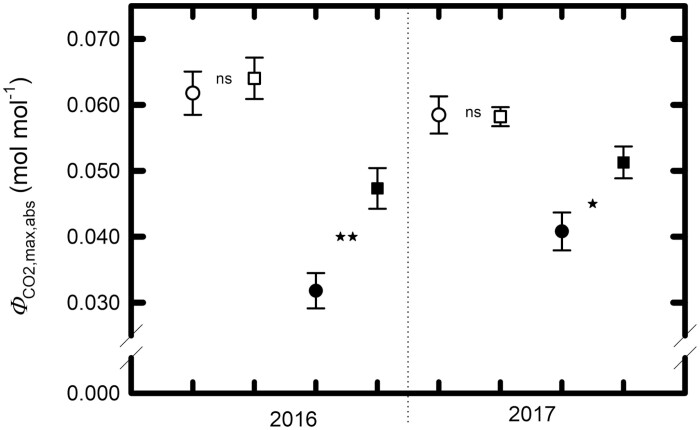
Mean maximum quantum yields of leaf CO_2_ uptake. Mean maximum quantum yields (±1 SE) of leaf CO_2_ uptake on an absorbed light basis (Φ _CO2,max,abs_) in sun leaves (open symbols) and shade leaves (filled symbols) of planophile and erectophile sorghum accessions, respectively. Means are based on eight planophile and 10 erectophile accessions in 2016, and nine planophile and 12 erectophile accessions in 2017, respectively. Symbols ‘*’ and ‘**’ represent statistical significance at α=0.05 and 0.01, respectively, when comparisons are made between erectophiles and planophiles at the same canopy position within the same year (ns, α>0.05). Data are from both field sites.

Consistent with our hypothesis, the reduction in quantum yield of carbon assimilation (Φ _CO2,max,abs_) in the lower canopy was greater in planophile than in erectophile accessions across the two years (*F*_12,143_=4.08, *P*=0.013: Fig. 3). In 2016, among the planophile accessions, Φ _CO2,max,abs_ dropped to 0.025–0.038 in the lower canopy, compared with 0.054–0.070 in the upper canopy (a 47% decrease), whereas, in the erectophile accessions, Φ _CO2,max,abs_ only decreased to 0.041–0.054 in the lower canopy, representing a 26% decrease (*t*=4.14, *P*=0.0012, df=13 for the type×position contrast). Similar effects were evident in 2017: in planophile accessions, Φ _CO2,max,abs_ was 30% lower in lower canopy leaves than in the upper canopy, while the corresponding decrease was only 12% in erectophiles (*t*=2.25, *P*=0.037, df=18 for the type×position contrast). When the leaf inclination angle was considered as a continuous variable, lower canopy quantum yield was negatively correlated with inclination angle in both 2016 (*r*^2^=0.72, *P*=0.0008) and 2017 (*r*^2^=0.30, *P*=0.0101). Light-adapted respiration, like quantum yield, also showed evidence of a decrease in the lower canopy that was modulated by shade intensity. In 2017, *R*_D_ in lower canopy leaves was 18% lower than for upper canopy leaves in erectophiles, but 52% lower in planophiles (*t*=2.31, *P*=0.033, df=7). Statistical analyses for Φ _CO2,max,abs_ and Φ _CO2,max,app_ results are given in Supplementary Table S5. Parameters extracted from light–response curves are presented in Supplementary Tables S6 and S7 for 2016 and 2017, respectively. Raw data from light–response curves are shown in Supplementary Tables S8 and S9 for 2016 and 2017, respectively.

In both years, light-saturated photosynthetic capacity (*A*_sat_) was much lower in the lower canopy. For example, in 2016, lower canopy rates were 12–21 µmol m^−2^ s^−1^, compared with 25–35 µmol m^−2^ s^−1^ in the upper canopy. As with respiration and quantum yield, this effect was less pronounced in erectophiles, with a 34% reduction versus 50% in planophiles (Table 1). The effect of shade intensity on modulating the effect of canopy position was therefore smaller for *A*_sat_ than for quantum yield. Although the convexity of the *A* versus *Q* light–response curve was significantly lower in the lower canopy, the difference between erectophiles and planophiles was not significant (Table 1).

**Fig. 4.**
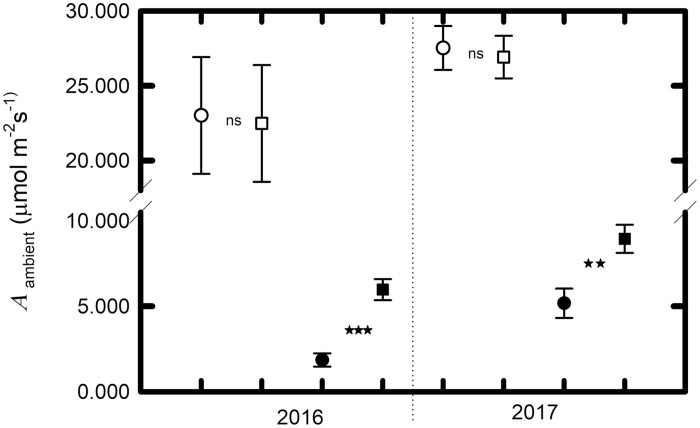
Mean maximum quantum yields of leaf CO_2_ uptake on an absorbed light basis. Mean leaf CO_2_ uptake (*A*_ambient_±1 SE) at the ambient light level in the field in sun leaves (open symbols) and shade leaves (filled symbols) of planophile and erectophile sorghum accessions, respectively. Means are for the same leaves as sampled in Fig. 3. Symbols ‘**’ and ‘***’ represent statistical significance at α=0.01 and 0.001, respectively, when comparisons are made between erectophiles and planophiles at the same canopy position within the same year (ns, α>0.05). Data are from both field sites.

**Fig. 5.**
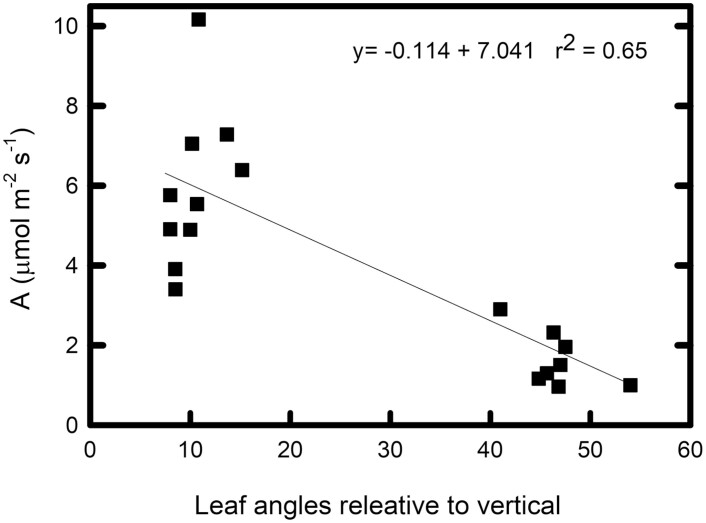
Carbon assimilation at ambient light level in 2016. Estimated carbon assimilation at ambient light level in the field (*A*_ambient_) as a function of leaf inclination angle (relative to the vertical) across 18 sorghum accessions; 10 erectophiles and eight planophiles for both field sites in 2016.

**Fig. 6.**
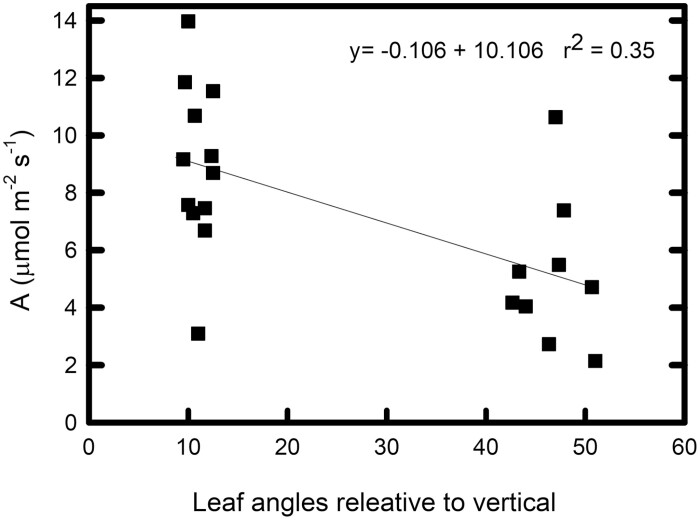
Carbon assimilation at ambient light level in 2017. Estimated carbon assimilation at ambient light level in the field (*A*_ambient_) as a function of leaf inclination angle (relative to the vertical) across 21 sorghum accessions; 12 erectophiles and nine planophiles for both field sites in 2017.

Because of the compounding effect of greater light availability, higher *A*_sat_ and higher Φ _CO2,max,app_, with a small offset from higher *R*_D_, lower canopy leaves of erectophile lines have substantially higher estimated carbon assimilation at midday on sunny days in mid-summer (*A*_ambient_: *F*_12,143_=18.81, *P*<0.0001). Based on field measurements of light availability in the upper and lower canopy, lower canopy leaves of erectophiles supported 3.2-fold greater carbon assimilation than lower canopy leaves in planophiles in 2016 (4.4–7.8 µmol m^−2^ s^−1^ versus 0.9–2.9 µmol m^−2^ s^−1^: *t*=6.38, *P*=0.0011, df=15: Fig. 4) and 72% more assimilation in 2017 (6.7–11.2 µmol m^−2^ s^−1^ versus 3.1–11.3 µmol m^−2^ s^−1^: *t*=3.25, *P*=0.0047, df=17; Fig. 4). Considering leaf inclination angle as a continuous variable, lower canopy estimated carbon assimilation (*A*_ambient_) was negatively correlated with leaf angle in both 2016 (*r*^2^=0.652, *P*<0.0001: Fig. 5) and 2017 (*r*^2^=0.343, *P*=0.586: Fig. 6). There was no significant difference in upper canopy assimilation between erectophiles and planophiles: therefore, expected levels of overall canopy-level photosynthesis would be higher in the erectophile accessions.

To quantify the whole-canopy difference in assimilation, we plotted each photosynthetic parameter (α, Φ _CO2,max,abs_, *R*_D_, and *A*_sat_) as a function of ambient light for (i) upper canopy leaves of both types; (iii) lower canopy leaves of erectophiles; and (iii) lower canopy leaves of planophiles. From these three points, we estimated linear relationships for each variable (*A*_sat_=0.0096*Q* + 16.04, *R*_D_=0.0017*Q*+0.843, Φ _CO2,max,app_=0.0001*Q* + 0.0399). θ was set at 0.5, since no position×type effect was detected (Table 1). The ambient light environment through the canopy was predicted (in increments of 0.01 of canopy depth) from the measured light extinction coefficients for July and August, assuming that leaves were evenly distributed through the canopy and that overall canopy LAI was 5.0, and these 100 increments were summed to obtain estimated whole-canopy photosynthesis (calculations shown in Supplementary Table S10). By counterfactually changing only the extinction coefficient and absorptance, and not changing the photosynthetic parameters (i.e. assuming no shade-related decline in leaf photosynthetic traits), the share of the decline in lower canopy assimilation due to the direct effect of less light in the lower canopy was projected. This allowed separation of any additional change due to the greater decline in Φ _CO2,max,abs_, *R*_D_, and *A*_sat_ in the planophile canopies. Based on these assumptions, planophile canopies would have 19% lower photosynthesis in July, with 83% of this decline due to direct effects of less light in the lower canopy and 17% due to the greater decline in photosynthetic capacity. In August, under heavier self-shading, the projected decline in planophiles relative to erectophiles was 21%, of which 89% was due to less light and 11% due to greater loss of photosynthetic capacity. In early season growth (June), self-shading would be less in both canopy types. Although measurements were not made in June, losses were projected assuming the same light-dependent changes observed in July and August. With assumed extinction coefficients of *k*=0.8 and 1.8 in erectophile and planophile canopies, respectively, planophiles would show 22% lower whole-canopy photosynthesis, with 64% of the decrease being due to lower light availability and 36% due to a greater decline in photosynthetic traits. This suggests that the greater loss of photosynthetic capacity in planophile canopies might have most impact in early season growth, but would be substantial throughout. A linear decline in assimilation from low to high leaf angle is presented (Figs 5, 6). However, our selection of the extremes in canopy types from the original results in two clusters, leaves some uncertainty in the assumption of linearity which would affect the calculated losses, but not their direction. Results of the full statistical analysis for *A*_ambient_ are in given in Supplementary Table S10, including erectophiles, planophiles, and counterfactual scenarios where only light environment or only photosynthetic traits, respectively, are changed. Light–response curves for upper and lower canopy leaves in 2016 and 2017 are shown graphically in Supplementary Figs S1–S4.

### Electron transport

The response of whole-chain electron transport to light (*J* versus *Q*) in both years showed less depression of quantum yield in the lower canopy than the *A* versus *Q* responses, and no significant difference between erectophiles and planophiles (Table 2). For example, in 2017, Φ _J_ in lower canopy leaves was 12–15% lower than in upper canopy leaves; this was equivalent to the modest decrease in Φ _CO2,abs_ in erectophile lower canopy leaves, and only half as large as the 30% decrease in the planophiles. The comparison of changes for electron transport and assimilation might indicate decoupling of electron transport and net CO_2_ assimilation under low light. Pooling across both years, the Φ _J_/Φ _CO2,abs_ ratio increased from 5.3–5.5 in the upper canopy to 6.0–6.3 in erectophile lower canopy leaves (*t*=2.51, *P*=0.023, df=17), but from 5.4–5.6 to 8.5–9.3 in planophiles (*t*=5.42, *P*<0.0001, df=16). This ratio is an index of how much reducing power is required to assimilate a molecule of CO_2_: therefore, such decoupling could potentially be linked to greater bundle sheath leakiness at low light or diversion of reductive power to sinks other than CO_2_ assimilation

**Table 2.** Means (±SE), Student’s *t*-values for planned contrasts, *r*^2^ values for correlation against leaf inclination angle, and *F*-values for quantum yield of electron transport under light-limited conditions (Φ _J_), maximal potential PSII efficiency at saturating light (*F*_V_′/*F*_M_′), ratio of quantum yield of electron transport to quantum yield of carbon assimilation (Φ _J_/Φ _CO2_), electron transport at ambient light levels (*J*_ambient_), and light-saturated electron transport rate (*J*_sat_) for sun leaves and shaded leaves of a set of erectophile (E) and planophile (P) sorghum accessions

Treatment	Φ_J_	*F*_V_′/*F*_M_′	Φ_J_/Φ_CO2_	*J*_ambient_	*J*_sat_
	mol electrons mol^−1^ light		–	μmol m^−2^ s^−1^	μmol m^−2^ s^−1^
P, Sun (2016)	0.358±0.008	0.337±0.007	5.98±0.18	121.3±16.2	134.7±20.1
P, Shade (2016)	0.329±0.027	0.308±0.014	11.88±0.93	29.0±3.1	127.2±16.0
Reduction (%)	8	8	–99	76	6
E, Sun (2016)	0.340±0.010	0.328±0.008	5.60±0.32	120.9±9.6	137.9±12.4
E, Shade (2016)	0.308±0.011	0.297±0.011	7.05±0.48***	56.9±4.3 ***	118.2±12.1
Reduction (%)	9	9	–26	53	14
P, Sun (2017)	0.294±0.009	0.303±0.010	5.09±0.14	171.9±10.0	202.6±17.4
P, Shade (2017)	0.251±0.017	0.266±0.021	6.20±0.24	31.7±4.9	115.0±12.4
Reduction (%)	15	12	-22	82	43
E, Sun (2017)	0.292±0.005	0.304±0.009	5.13±0.12	169.7±6.0	200.4±10.0
E, Shade (2017)	0.258±0.008	0.296±0.008	5.40±0.25*	60.4±6.6 **	147.0±10.9
Reduction (%)	12	3	–5	64	35
**Correlation**					
**2016**	0.001	0.000	0.652***	0.636***	0.031
**2017**	0.000	0.091	0.265*	0.348**	0.168
Position	–4.23***	–3.85**	5.11***	–13.48***	4.13***
Type×Position	–0.13	–0.99	–3.59**	–3.49**	–0.32
**Source of variation**					
	*F*_12,143_	*F*_12,143_	*F*_12,143_	*F*_12,143_	*F*_12,143_
Environment	9.03***	2.24	2.13	10.92***	7.15***
Type	0.86	0.16	24.93***	15.61***	0.21
Height	4.42*	0.02	1.01	2.80	0.35
Env×Type	1.79	0.60	0.41	0.17	0.08
Position	11.04***	5.84**	35.36***	162.99***	12.03***
Type×Position	0.17	0.94	13.01***	8.40***	0.76

*J*_ambient_ was measured at ambient light levels for each leaf, at midday on a clear sky day in August; Φ _J_ and Φ _J_/Φ _CO2_ were measured over the light-limited range of photosynthesis (0–150 μmol m^−2^ s^−1^), and *F*_V_′/*F*_M_′ and *J*_max_ were measured at saturating light. Samples and statistical symbols are as in Table 1.

Maximal electron transport capacity (*J*_sat_) was higher in sun leaves than in shade leaves (132–161 μmol m^−2^ s^−1^ versus 83–111 μmol m^−2^ s^−1^), but there were no differences between erectophiles or planophiles in either *J*_sat_ or the response of *J*_sat_ to canopy position (i.e. no interaction between canopy position and canopy type: Table 2). The convexity of the electron transport versus light curve (θ _J_) was unaffected by light (Supplementary Table S3). As noted for gas exchange, electron transport-related parameters extracted from light–response curves are presented in Supplementary Tables S6 and S7 for 2016 and 2017, respectively, while raw data are shown in Supplementary Tables S8 and S9 for 2016 and 2017, respectively.

### Water use efficiency

Neither the initial slope nor the convexity of the stomatal conductance versus light curve was affected by canopy position or canopy structure (Table 1). Light-saturated maximal conductance (*g*_S,sat_) was higher in upper canopy leaves than in lower canopy leaves. However, this effect of canopy position was similar in planophiles and erectophiles. The combination of higher assimilation and similar stomatal conductance, in lower canopy leaves of erectophiles compared with planophiles, resulted in a large advantage for erectophiles in IWUE_ambient_ (Table 1). While upper canopy leaves of both forms had similar intrinsic water use efficiency, IWUE_ambient_ was >50% higher in lower canopy leaves of erectophiles compared with planophiles (168–188 μmol mol^−1^ versus 105–121 μmol mol^−1^: *t*=3.89, *P*=0.002, df=13).

### Bundle sheath leakiness

Bundle sheath leakiness, among the 12 accessions surveyed in summer 2020, showed no change with descending canopy position, when measured either at high or at low irradiance (Table 3). Consistent with previous findings (e.g. Kromdijk *et al.*, 2008), leakiness was much higher at low irradiance: when each leaf was measured at 2200 μmol m^−2^ s^−1^ and at 80 μmol m^−2^ s^−1^, ϕ at low light intensity was consistently higher (0.39–0.46 versus 0.27–0.31).

**Table 3.** Means (±SE), *r*^2^ values for correlation against leaf inclination angle, Student’s *t*-values for planned contrasts, and *F*-values for carbon assimilation (*A*), stomatal conductance (*g*_S_), and bundle sheath leakiness (ϕ), measured at two levels of light (2200 μmol m^−2^ s^−1^ and 80 μmol m^−2^ s^−1^) in sun leaves and shaded leaves of a set of six erectophile (E) and six planophile (P) sorghum accessions

Treatment	*A*	*g*_S_	ϕ	*A*	*g*_S_	ϕ
	μmol m^−2^ s^−1^	mol m^−2^ s^−1^	–	μmol m^−2^ s^−1^	mol m^−2^ s^−1^	–
Photon flux:	High	High	High	Low	Low	Low
P, Sun	38.40±2.38	0.235±0.014	0.267±0.013	1.69±0.20	0.036±0.011	0.386±0.016
P, Shade	26.95±3.46	0.160±0.022	0.314±0.011	2.04±0.10	0.058±0.037	0.467±0.028
Reduction (%)	30	32	–18	–20	–54	–21
E, Sun	36.91±1.76	0.260±0.028	0.270±0.011	1.38±0.22	0.081±0.035	0.438±0.050
E, Shade	22.90±3.29	0.162±0.038	0.302±0.018	1.44±0.18	0.020±0.004	0.431±0.038
Reduction (%)	38	38	-12	-4	75	2
**Correlation**						
Upper canopy	0.029	0.001	0.000	0.006	0.001	0.001
Lower canopy	0.128	0.000	0.000	0.016	0.094	0.001
**Contrast**						
Level	–3.04**	–3.04*	1.79	0.96	–0.49	0.62
Type×Position	0.42	0.54	–0.47	–0.96	1.46	–1.06
**Source of variation**						
	*F*_14,9_	*F*_14,9_	*F*_14,9_	*F*_14,9_	*F*_14,9_	*F*_14,9_
Type	0.01	1.07	0.59	4.51	1.78	0.41
Position	9.19*	11.26**	2.83	8.50*	4.18	0.18
Type×Position	0.26	0.16	0.00	5.84*	1.30	0.35

Correlation (*r*^2^) values refer to the correlation of parameter values against leaf angle, for both upper and lower canopy leaves. Data are from a summer 2020 field study in Urbana, IL. For cell means, symbols ‘*’, ‘**’, and ‘***’ denote statistical significance at a two-tailed α=0.05, 0.01, and 0.001, respectively, when erectophiles are compared against planophiles at the same canopy position and within the same year. For *F*-values, *r*^2^ values, and planned contrasts, symbols ‘*’, ‘**’, and ‘***’ denote statistically significant effects of an explanatory variable at a two-tailed probability α=0.05, 0.01, and 0.001 respectively.

### Productivity

Across 2016 and 2017, biomass was affected by height (*F*_1,35_=9.68, *P*=0.004), by canopy type (*F*_1,35_=23.58, *P*<0.001), and by sampling year (*F*_1,35_=36.16, *P*<0.001). Erectophile accessions produced 33% more biomass than planophiles—a 6.5 Mg ha^−1^ difference (25.55±1.26 Mg ha^−1^ versus 19.07±1.47 Mg ha^−1^: *t*=2.53, *P*=0.017, df=31). Effects of leaf erectness on productivity were also present when leaf angle was considered as a continuous rather than a categorical variable: leaf angle was also negatively correlated with biomass in both 2016 (*r*^2^=0.37, *P*=0.0036) and 2017 (*r*^2^=0.39, *P*=0.0026). Biomass data are shown in (Supplementary Tables S11 and S12 for 2016 and 2017, respectively.

### C_4_ photosynthetic enzymes

In 2017, all three of the C_4_ cycle enzymes assayed showed clear trends, of comparable magnitude, towards lower activity in shade leaves; however, there was no evidence of differing shade responses in erectophile and planophile accessions (Table 4; (Supplementary Table S13). Maximal extractable PPDK activity in the 2017 samples was 30% lower in shade leaves than in sun leaves, while the *V*_max_ of PEPC was 36% lower and that of MDH was 39% lower.

**Table 4.** Means (±SE) and *F*-values for maximal *in vitro* extractable activity (*V*_max_) of pyruvate orthophosphate dikinase (PPDK), phosphoenolpyruvate carboxylase (PEPC), and NADP-dependent malate dehydrogenase (MDH), as well as Rubisco content, in sun leaves and shaded leaves of a set of erectophile accessions (small leaf inclination angle relative to vertical and low self-shading, denoted ‘E’) and planophile accessions (large leaf inclination angle relative to vertical and high self-shading, denoted ‘P’) of *Sorghum bicolor*

Treatment×Shade	PPDK	PEPC	MDH	Rubisco
	µmol m^−2^ s^−1^	µmol m^-2^ s^−1^	µmol m^−2^ s^−1^	µg mg^−1^
P, Sun	49.3±9.4	453.2±67.4	104.4±11.1*	2.45±0.31
P, Shade	37.4±4.1	304.8±49.7	56.7±14.8	2.35±0.20
Reduction (%)	24	33	46	22
E, Sun	50.0±6.4	418.7±61.6	70.9±17.8	2.57±0.17
E, Shade	33.2±4.5	263.3±22.0	45.4±14.0	2.30±0.22
Reduction (%)	34	37	36	80
Position	–2.79*	–2.97*	3.09*	0.69
Type×Position	0.71	-0.04	–0.96	0.33
**Source of variation**				
	*F*_21,13_	*F*_21,13_	*F*_21,13_	
Type	0.26	0.35	0.00	0.14
Height	0.89	0.60	0.27	–
Position	10.38**	9.69**	10.87**	0.05
Type×Position	0.04	0.04	1.21	0.58

Activity data for PPDK, PEPC, and MDH were based on samples collected in early September 2017 (11 erectophile and nine planophile accessions): content data for Rubisco were based on samples collected in mid-August 2020 (12 erectophile and 12 planophile accessions). MDH was assayed after incubation with a high concentration of DTT to fully activate the enzyme. Activity measurements were done at 30 °C. For cell means, symbols ‘*’, ‘**’, and ‘***’ denote statistical significance at a two-tailed α=0.05, 0.01, and 0.001, respectively, when erectophiles are compared against planophiles at the same canopy position, For *F*-values, symbols ‘*’, ‘**’, and ‘***’ denote statistically significant effects of an explanatory variable at a two-tailed α=0.05, 0.01, and 0.001, respectively. Data for MDH, PEPC, and PPDK are from a 2016–2020 field study (Urbana, IL).

In contrast to the C_4_ cycle enzymes, the samples collected in 2020 showed no trend towards lower Rubisco content in the shade, or any difference between erectophiles and planophiles (Table 4; Supplementary Table S14).

## Discussion

As hypothesized, our data indicate that the maximum quantum efficiency of CO_2_ uptake (Φ _CO2,abs_), the key measure of photosynthetic efficiency in limiting light, declined in a maladaptive way into the lower canopy across 35 accessions of *S. bicolor*. However, the decline was much greater in planophile canopies, where the lowest fully green leaves received 5–10% of full sunlight, than in erectophile canopies, where the lowest corresponding leaves received 15–24% of full sunlight (Table 1; Fig. 2). The agronomic significance of the inability to acclimate to very low light environments is implicit in our study. Increasing leaf angles in cereals, including sorghum, has been associated with increased yields (Sinclair and Sheehy, 1999; Lee and Tollenaar, 2007; DaSilva and DeCosta, 2012; Lauer *et al.*, 2012; Richards *et al.*, 2019). This is largely because upper leaves in planophile canopies intercept more light than can be utilized in photosynthesis, while photosynthesis in the lower leaves is strongly light limited: erectophile forms therefore allow a more effective distribution of light. Here, in the case of sorghum, and possibly other crops of the Andropogoneae (Pignon *et al.*, 2017; Collison *et al.*, 2020), we show a second beneficial effect: maintenance of light-limited photosynthetic capacity and water use efficiency in the lower canopy leaves (Fig. 2). The fact that C_4_ enzymes accounting for a significant amount of leaf soluble nitrogen were at similar levels in the lower canopy of planophiles and erectophiles (Table 4) suggests that nitrogen use efficiency might also be a further benefit of erectophile canopies. Here, leaf angle was only measured at the point of insertion into the stem. Sorghum leaves bow and so the angle will show a progressively larger angle with distance from the stem. Our assumption was that a low leaf angle at the point of insertion would result in lower angles across the leaf. That light reaching the lowest leaves in the erectophile canopies was double that of the planophile canopies, as judged by the angle at the point of insertion, suggests there was no compensatory effect, namely those with small angles at the point of insertion showing greater bowing (Fig. 2).

By comparing a wide range of germplasm, our results indicate that declines in quantum efficiency of carbon assimilation under self-shading are a species-wide phenomenon in sorghum, not limited to one or a few genotypes. In addition, much of the previous literature on sun versus shade acclimation has considered leaves grown under continuous high versus low light. Our study is relatively unusual in that it documents effects on Φ _CO2,max,app_ when C_4_ leaves that emerge in high-light conditions are progressively subjected to increasingly heavy shade, a situation relevant to many agricultural and grassland environments. Previous studies found that self-shading in lower canopy leaves of *Miscanthus* and maize led to declines in Φ _CO2,max,app_, contrasting sharply with some C_3_ crops such as wheat (Kromdijk *et al.*, 2008; Pignon *et al.*, 2017; Collison *et al.*, 2020). However, these studies considered only single genotypes; our data extend these previous studies by showing that quantum yield declines are a broadly observed pattern in sorghum, and are related to the degree of self-shading. Given that this has now been observed in three species of Andropogoneae, it suggests that this maladaptive response might apply to the full tribe.

The decline in Φ _J_ with canopy depth was much smaller than for Φ _CO2_ and was not affected by the degree of self-shading. This may indicate that increases in oxidative stress, non-photochemical quenching, or inefficiencies in carbon assimilation, leading to decoupling between electron transport and assimilation, could be part of the explanation for the decline in quantum yield under shading. However, increases in bundle sheath leakiness (ϕ) appear not to be the explanation for changes in Φ _J_/Φ _CO2_, since canopy position did not affect ϕ when sun and shade leaves were compared at the same light intensity. The lack of shade-induced increase in ϕ was consistent with the fact that Rubisco content showed much less of a decrease (not statistically significant) in lower canopy leaves than the mesophyll cycle photosynthetic enzymes, indicating that Rubisco in the bundle sheath should be able to keep up with the influx of CO_2_ from the mesophyll. Previous literature has shown that bundle sheath leakiness increases with canopy depth in giant *Miscanthus,* from 0.2–0.5 under high light to 0.8 at the lowest light levels (Kromdijk *et al.*, 2008), while similar effects on leakiness are seen in maize (Kromdijk *et al.*, 2010), *Amaranthus* (Tazoe *et al.*, 2008), and *Flaveria* (Pengelly *et al.*, 2010). However, these effects seem to be purely the result of lower ambient light, and not an acclimatory response. Bellasio and Griffiths (2014) grew young maize plants of a single cultivar in high light and low light in controlled-environment cabinets. In parallel with the results here, ϕ was almost doubled in low light compared with high light (cf. Table 3). However, Bellasio and Griffiths found a slightly lower ϕ in the plants grown in low light, at all measurement light intensities, suggesting some acclimation. Here a significant difference between leaves in low light and high light was not found across 12 accessions.

In contrast to quantum yield and respiration rates, erectophile accessions showed similar declines in activity of C_4_ enzymes to the planophiles. Activities of PPDK and MDH can co-limit the light-saturated photosynthetic rate, and therefore a decrease in the activity of these enzymes in lower canopy leaves is consistent with the observed decline in *A*_sat.._ The observed decreases in activity of C_4_ enzymes, in the lower canopy, are also consistent with previous studies of C_4_ plants grown under continuous high or low light (Sonawane *et al.*, 2018). Unlike the declines in Φ _CO2_, these changes are likely to be adaptive. Since these parameters influence the maximum light-saturated rate of photosynthesis, decreased levels of the C_4_-specific enzymes and of light-saturated photosynthetic capacity would be advantageous, allowing resources to be relocated to the upper leaves.

Breeders have long recognized that in this highly productive group of C_4_ crops (Andropogoneae) erect leaf angle phenotypes are associated with elevated grain yields (Lee and Tollenaar, 2007; DaSilva and DeCosta, 2012; Lauer *et al.*, 2012; Lofton *et al.*, 2012; Zhang *et al.*, 2014; Truong *et al.*, 2015). These effects have been attributed entirely to a more effective distribution of light in the canopy (Gitz *et al.*, 2015). Likewise, previous modeling work has predicted the effect of reducing leaf angle through selection or synthetic biology on sorghum productivity, but has typically assumed large changes in light interception coupled with unchanged leaf photosynthetic traits ( Gitz *et al.*, 2015; Truong *et al.*, 2015). This study identifies a different mechanism by which the erectophile form gains an important advantage, and indicates that the advantage of erectophile canopies could be even greater than previous models have predicted. The decline in capacity to assimilate CO_2_ in the low-light conditions of the lower crop canopy appears an underappreciated explanatory factor for previously documented observational data. While the erectophile form reduces this loss, it is still present. Here all accessions were planted at the same density. However, if more erect form cultivars are grown at higher densities, the loss in efficiency seen here would be exacerbated. Understanding the mechanism, such that it might be eliminated, would lead to greater yield gains, especially if trends toward increasing planting density continue.

## Supplementary data

The following supplementary data are available at *JXB* online.

Table S1. Leaf angles for 869 sorghum accessions in summer 2016.

Table S2. Leaf angles for 869 sorghum accessions in summer 2017.

Table S3. Parameters extracted from light profiles in erectophile and planophile sorghum canopies in summer 2018.

Table S4. Photosynthetically active radiation as a function of depth through the canopy in erectophile and planophile sorghum accessions in summer 2020.

Table S5. Means, SEs, and *F-*values from ANOVA for quantum yield of carbon assimilation, estimated carbon assimilation under ambient light conditions, and convexity of the electron transport curve for erectophile and planophile sorghum canopies.

Table S6. Parameters extracted from photosynthetic light–response curves for upper and lower canopy leaves of erectophile and planophile sorghum accessions, collected in summer 2016.

Table S7. Parameters extracted from photosynthetic light–response curves for upper and lower canopy leaves of erectophile and planophile sorghum accessions, collected in summer 2017.

Table S8. Raw data for light–response curves collected in summer 2016.

Table S9. Raw data for light–response curves collected in summer 2017.

Table S10. Predicted carbon assimilation at various increments of depth through the canopy, in erectophile and planophile sorghum accessions, under various hypothetical scenarios assuming changes in light penetration, changes in leaf-level photosynthetic traits, or both.

Table S11. Height and biomass data for selected erectophile and planophile sorghum accessions in summer 2016.

Table S12. Height and biomass data for selected erectophile and planophile sorghum accessions in summer 2016.

Table S13. *In vitro* activity of PEP carboxylase, pyruvate phosphate dikinase, and malate dehydrogenase in upper and lower canopy leaves of erectophile and planophile sorghum accessions in summer 2017.

Table S14. Rubisco content in upper and lower canopy leaves from erectophile and planophile sorghum accessions in summer 2020.

Fig. S1. Carbon assimilation (*A*_sat_) as a function of incident light (*I*), along with fitted curves, in upper and lower canopy leaves of 10 sorghum accessions with highly erect leaves (erectophile canopy structure) in a 2016 field experiment.

Fig. S2. Carbon assimilation (*A*_sat_) as a function of incident light (*I*), along with fitted curves, in upper and lower canopy leaves of eight sorghum accessions with drooping leaves (planophile canopy structure) in a 2016 field experiment.

Fig. S3. Carbon assimilation (*A*_sat_) as a function of incident light (*I*), along with fitted curves, in upper and lower canopy leaves of 12 sorghum accessions with highly erect leaves (erectophile canopy structure) in a 2017 field experiment.

Fig. S4. Carbon assimilation (*A*_sat_) as a function of incident light (*I*), along with fitted curves, in upper and lower canopy leaves of nine sorghum accessions with drooping leaves (planophile canopy structure) in a 2017 field experiment.

erab176_suppl_Supplementary_Figures

erab176_suppl_Supplementary_Table-S5

erab176_suppl_Supplementary_Table-S1-S14

## Data Availability

All original data that support the findings of this study are freely available through the Illinois Data Bank https://doi.org/10.13012/B2IDB-4580996_V1
