# Supplementary material for: Can improved canopy light transmission ameliorate loss of photosynthetic efficiency in the shade? An investigation of natural variation in Sorghum bicolor
Source: J Exp Bot. 2021 Apr 29;72(13):4965–80. doi: 10.1093/jxb/erab176 (PMC8219039; doi:10.1093/jxb/erab176)
Supplement: erab176_suppl_Supplementary_Figures [file erab176_suppl_supplementary_figures.pdf]

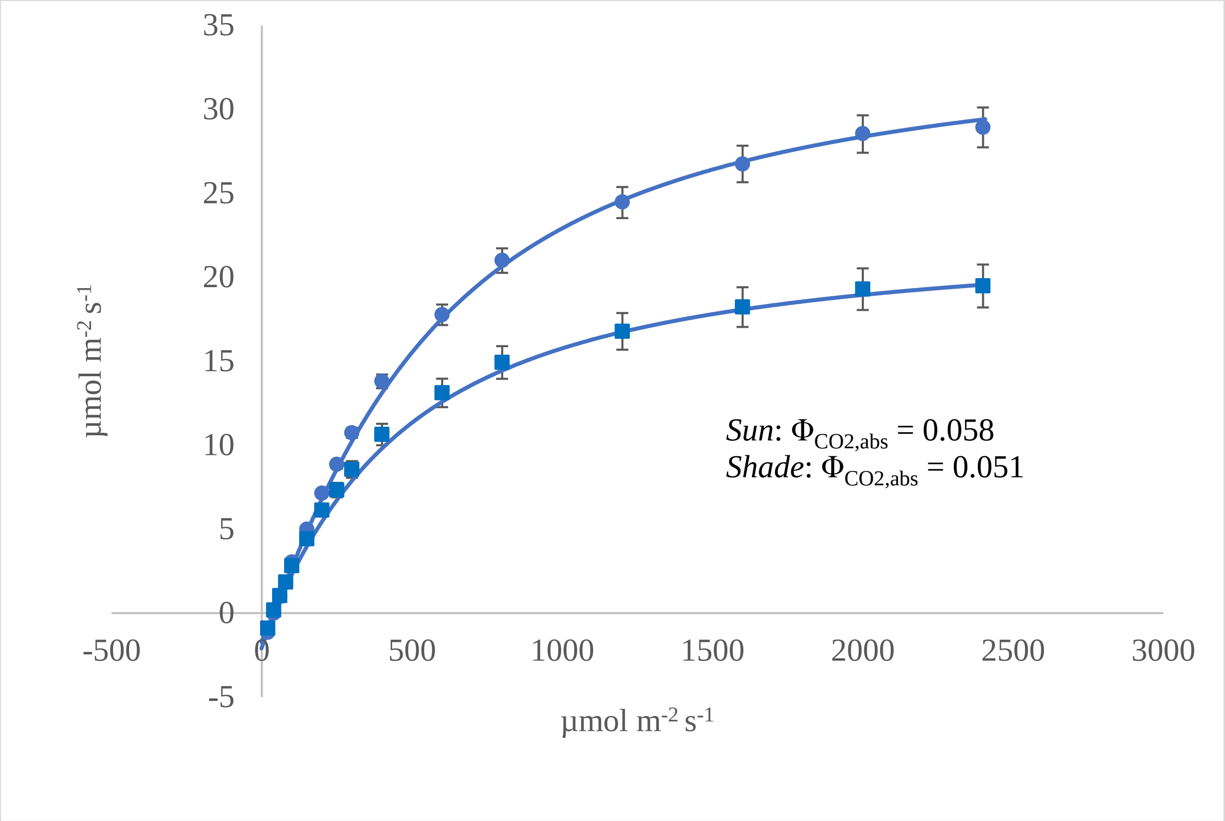

Supplementary Fig. S1. Carbon assimilation (A_{sat}) as a function of incident light (I), along with fitted curves, in upper canopy leaves (triangles) and lower canopy leaves (circles) of 10 sorghum accessions with highly erect leaves (erectophile canopy structure). Photosynthetic measurements were taken on cut leaves in late summer 2016, as part of a 2016-2018 study on the effect of self shading on leaf photosynthetic traits. Each accession was replicated at two sites (Savoy, IL and Urbana, IL respectively).

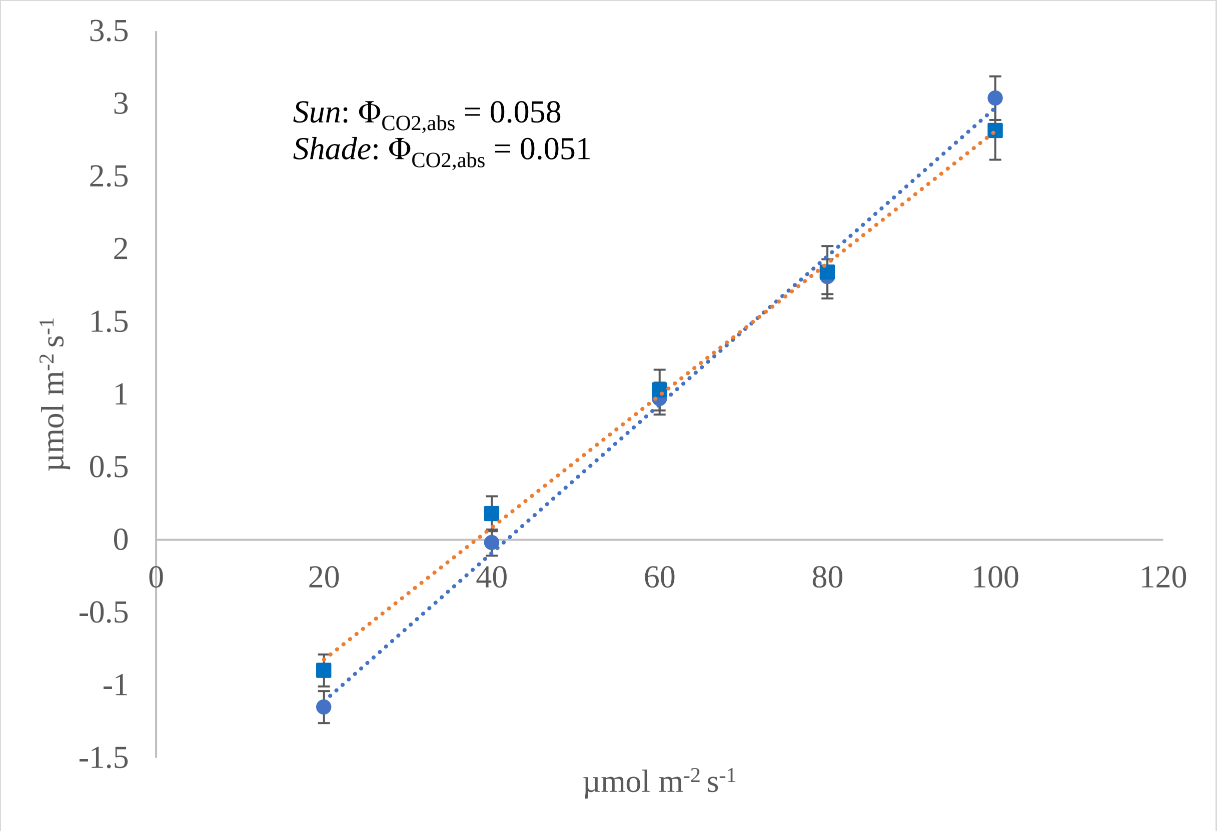

Supplementary Fig. S2. Carbon assimilation (A_{sat}) as a function of incident light (I), along with fitted curves, in upper canopy leaves (triangles) and lower canopy leaves (circles) of 8 sorghum accessions with drooping leaves (planophile canopy structure). Photosynthetic measurements were taken on cut leaves in late summer 2016, as part of a 2016-2018 study on the effect of self shading on leaf photosynthetic traits. Each accession was replicated at two sites (Savoy, IL and Urbana, IL respectively).

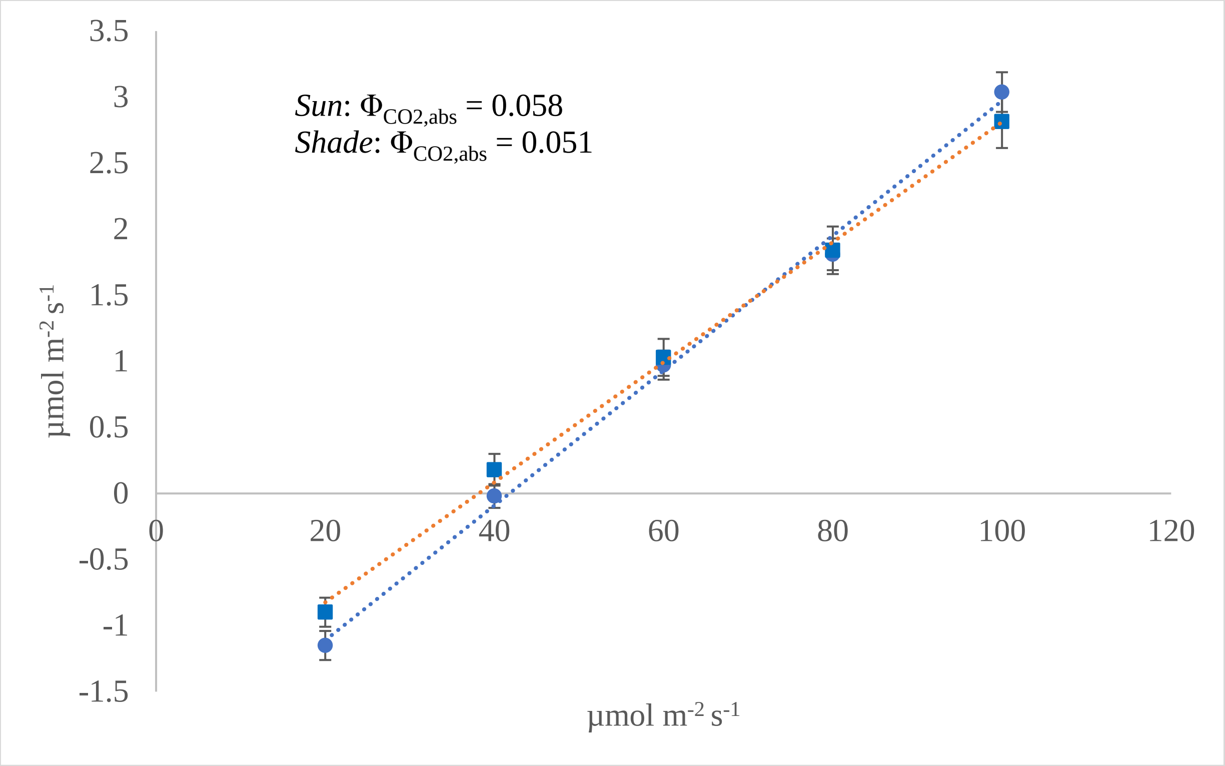

Supplementary Fig. S1. Carbon assimilation (A_{sat}) as a function of incident light (I), along with fitted curves, in upper canopy leaves (triangles) and lower canopy leaves (circles) of 12 sorghum accessions with highly erect leaves (erectophile canopy structure). Photosynthetic measurements were taken on cut leaves in late summer 2017, as part of a 2016-2018 study on the effect of self shading on leaf photosynthetic traits. Each accession was replicated at two sites (Savoy, IL and Urbana, IL respectively).

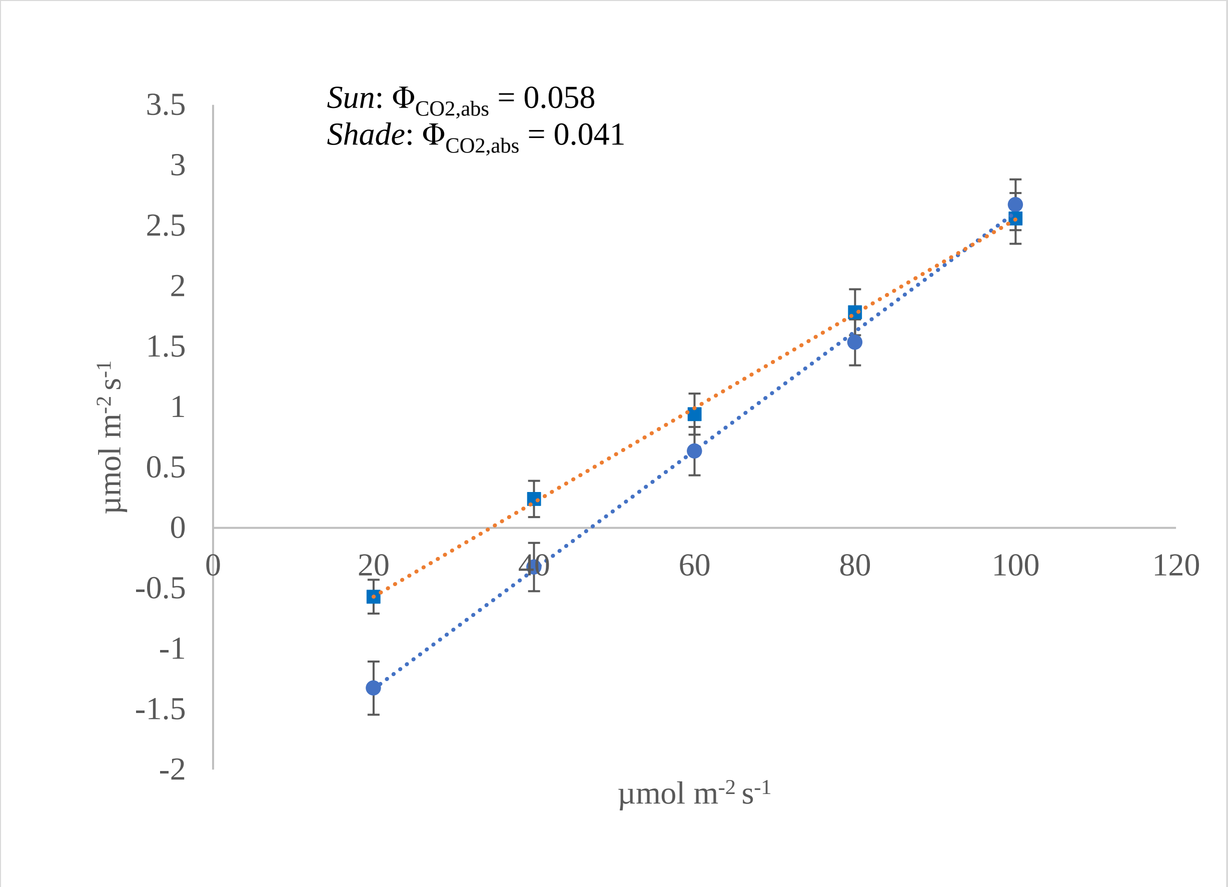

Supplementary Fig. S1. Carbon assimilation (A_{sat}) as a function of incident light (I), along with fitted curves, in upper canopy leaves (triangles) and lower canopy leaves (circles) of 9 sorghum accessions with drooping leaves (planophile canopy structure). Photosynthetic measurements were taken on cut leaves in late summer 2017, as part of a 2016-2018 study on the effect of self shading on leaf photosynthetic traits. Each accession was replicated at two sites (Savoy, IL and Urbana, IL respectively).
