# Supplementary material for: Can improved canopy light transmission ameliorate loss of photosynthetic efficiency in the shade? An investigation of natural variation in Sorghum bicolor
Source: J Exp Bot. 2021 Apr 29;72(13):4965–80. doi: 10.1093/jxb/erab176 (PMC8219039; doi:10.1093/jxb/erab176)
Supplement: erab176_suppl_Supplementary_Table-S5 [file erab176_suppl_supplementary_table-s5.pdf]

Table S5. Means (± standard error), r^2 values for correlation against leaf inclination angle, Student's *t*-values for planned contrasts, and *F*-values from analysis of variance, for quantum yield of carbon assimilation on absorbed-light basis ($\Phi_{CO2,max,abs}$), quantum yield of carbon assimilation on incident light basis ($\Phi_{CO2,max,app}$), estimated carbon assimilation under ambient light levels in the field ($A_{ambient}$), estimated electron transport under ambient light levels in the field ($J_{ambient}$), and convexity of the electron transport curve (θ_J) for sun leaves and shaded leaves of erectophile and planophile sorghum accessions. Quantum yields were measured under light-limited conditions (0 – 150 µmol m^{-2} s^{-1}), while $A_{ambient}$ and $J_{ambient}$ were measured under ambient light levels measured in the field on a sunny day (at midday) in mid-August. Erectophile accessions have ssmall leaf inclination angle relative to vertical (denoted "E"), and planophile accessions have large leaf inclination angle relative to vertical (denoted "P"). Eight planophile and ten erectophile lines were measured in 2016; nine planophile and 12 erectophiles were measured in 2017. Data are from a 2016-2017 field study. In both years, each accession was replicated at two field sites (Savoy, IL and Urbana, IL). For cell means, symbols '*', '**', and '***' denote statistical significance at a two-tailed α = 0.05, 0.01, and 0.001 respectively, when contrasts are made between erectophiles and planophiles at the same canopy position and within the same year. For *t*-values, r^2 values and *F*-values, symbols '*', '**', and '***' denote statistically significant effects of an explanatory variable at a two-tailed α = 0.05, 0.01, and 0.001 respectively.

**Treatment**	$\Phi_{CO2,max,\ abs}$ mol mol^{-1}	$\Phi_{CO2,max,app}$ mol mol^{-1}	$A_{ambient}$ µmol m^{-2} s^{-1}	Θ_J ---
P – Sun (2016)	0.062 ± 0.003	0.052 ± 0.003	23.0 ± 1.9	0.66
P – Shade (2016)	0.031 ± 0.003	0.028 ± 0.002	1.6 ± 0.2	0.70
Reduction (%)	49	46	93	-5
E-Sun (2016)	0.064 ± 0.003	0.054 ± 0.003	22.4 ± 1.2	0.79
E-Shade (2016)	0.047 ± 0.003**	0.041 ± 0.003**	6.0 ± 0.6***	0.79
Reduction (%)	26	25	73	-1
P – Sun (2017)	0.058 ± 0.003	0.050 ± 0.002	27.5 ± 1.5	0.81
P – Shade (2017)	0.041 ± 0.003	0.036 ± 0.003	5.2 ± 0.9	0.86
Reduction (%)	30	26	81	-5
E – Sun (2017)	0.058 ± 0.001	0.049 + 0.001	26.9 ± 1.4	0.78
E – Shade (2017)	0.051 ± 0.002*	0.044 + 0.002*	8.9 ± 0.8**	0.82
Reduction (%)	12	10	67	-6

**Correlation**				
**2016**	0.513***	0.468**	0.652***	0.046
**2017**	0.300*	0.248*	0.344**	0.026
**Contrast**				
Position	-9.07***	-9.73***	-17.25***	1.02
Type x Position	-3.83***	-3.73***	-4.76***	0.02
**Source of Variation**				
	F	*F*	*F*	*F*
Environment	1.26	1.26	4.28**	2.39
Type	10.12**	12.33***	33.26***	1.08
Height	0.37	0.39	0.16	0.21
Env x Type	0.48	0.47	0.13	1.67
Position	36.32***	42.16***	402.08***	0.51
Type x Position	4.08*	4.24*	18.81***	0.05
